# Demonstrating a Novel, Hidden Source of Spectral Distortion in X-ray Photon Counting Detectors and Assessing Novel Trigger Schemes Proposed to Avoid It

**DOI:** 10.3390/s23094445

**Published:** 2023-05-01

**Authors:** Oliver L. P. Pickford Scienti, Dimitra G. Darambara

**Affiliations:** Joint Department of Physics, Institute of Cancer Research and Royal Marsden NHS Foundation Trust, London SM2 5NG, UK; dimitra.darambara@icr.ac.uk

**Keywords:** energy resolving, photon counting, spectral imaging, CdTe, negative counts, count-triggering schemes

## Abstract

X-ray photon counting spectral imaging (x-CSI) determines a detected photon’s energy by comparing the charge it induces with several thresholds, counting how many times each is crossed (the standard method, STD). This paper is the first to demonstrate that this approach can unexpectedly delete counts from the recorded energy spectrum under some clinically relevant conditions: a process we call negative counting. Four alternative counting schemes are proposed and simulated for a wide range of sensor geometries (pixel pitch 100–600 µm, sensor thickness 1–3 mm), number of thresholds (3, 5, 8, 24 and 130) and medically relevant X-ray fluxes (10^6^–10^9^ photons mm^−2^ s^−1^). Spectral efficiency and counting efficiency are calculated for each simulation. Performance gains are explained mechanistically and correlated well with the improved suppression of “negative counting”. The best performing scheme (Shift Register, SR) entirely eliminates negative counting, remaining close to an ideal scheme at fluxes of up to 10^8^ photons mm^−2^ s^−1^. At the highest fluxes considered, the deviation from ideal behaviour is reduced by 2/3 in SR compared with STD. The results have significant implications both for generally improving spectral fidelity and as a possible path toward the 10^9^ photons mm^−2^ s^−1^ goal in photon-counting CT.

## 1. Introduction

**Figure 1:**
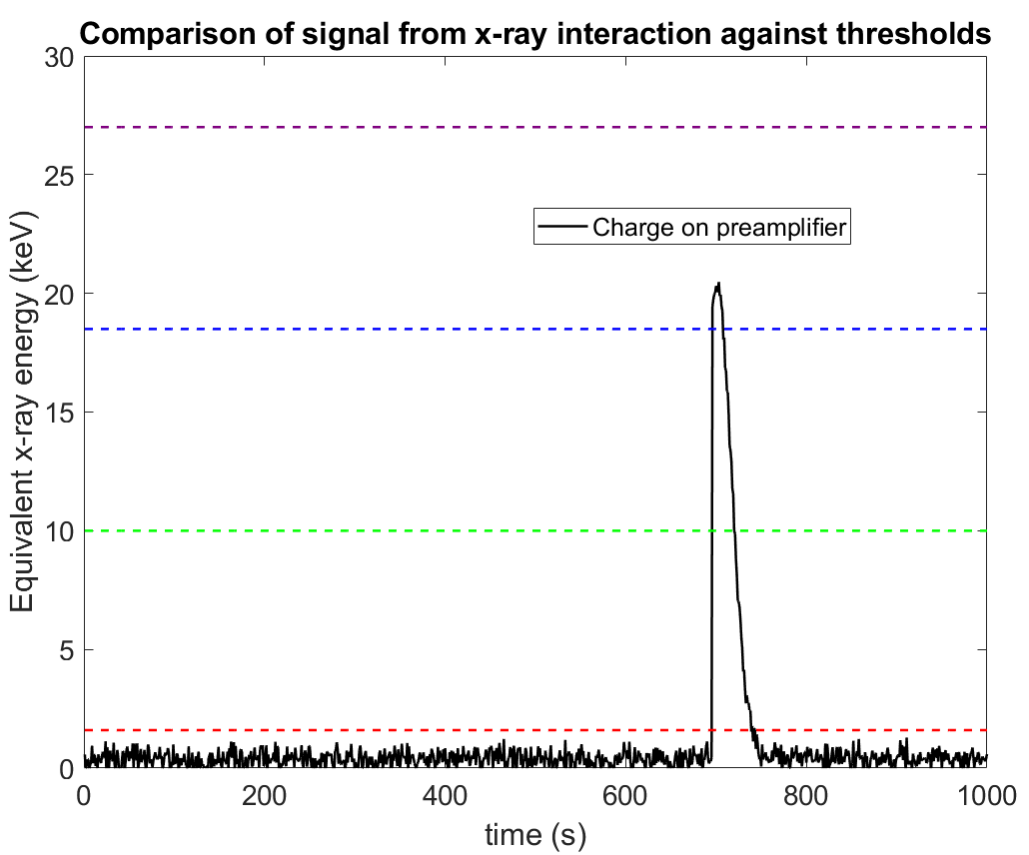
Photon counting works by comparing the signal out of the sensor (in this case the charge on the preamplifier, black line) against a pre-set threhsold (dashed lines). A threshold (red dashed line) is set so as to be several standard deviations from the mean of the electronic noise so false counts from noise are extremely unlikely. In an x-CSI system, additional thresholds are employed (green, blue and purple dashed lines), so that the energy of the photon can be more accurately estimated. In this instance, the incoming photon can be seen to be at some energy between 18.5 and 27 keV (the blue and purple threhsolds respectively).

X-ray photon counting (PC) approaches are widely accepted as the next advance in X-ray imaging, with the first clinical system now approved by the Food and Drug Administration (FDA) and several such systems currently under development by major medical manufacturers [1,2,3,4,5]. In contrast with conventional energy integrating (EI) X-ray detectors, which measure the total energy deposited within a pixel during an exposure, PC aims to measure the number of photons which interact with a pixel within the acquisition. To do this requires a much higher timing resolution, as each X-ray photon needs to be resolved individually. Broadly speaking, this is done by allowing the charge in each pixel to bleed off during the image acquisition via a preamplifier throughout the X-ray acquisition, and continuously assessing the charge on that preamplifier against a pre-set threshold (Figure 1). When the charge rises above the threshold, a counter associated with that threshold is incremented [6]. An advantage of PC is that electronic noise can be largely stripped out of the acquired images, in stark contrast with EI approaches which integrate electronic noise into the signal. PC achieves this by placing the counting threshold several standard deviations above the mean of the electronic noise, making the probability that electronic noise alone causes the charge to rise above the threshold negligible.

PC approaches are favoured in X-ray imaging for a variety of reasons: the images have a lower noise level [7]; they offer higher dose efficiencies (meaning lower patient doses can be used) [8,9]; they often offer higher spatial resolutions (as the pixel can be smaller when electronic noise is not a concern) [10]; they provide truly quantitative images [11,12]; and they improve soft tissue contrast (due to the equal weighting of low- and high-energy photons) [8]. The real advantages of PC can be realized by extending the technique from photon counting to energy discriminating by including multiple charge threshold–counter pairs. Figure 1 shows how this approach can be used to separate incoming X-ray photons based on their energy, providing spectral information from a single acquisition. This additional information allows for material decomposition tasks to be performed without the need for multiple sources or detectors. This approach is often referred to as spectral-photon-counting-computed tomography (SPCCT) [13,14] or X-ray photon counting spectral imaging (x-CSI) [15]. We will use the term x-CSI in this paper, to refer to the approach as applied more generally to X-ray imaging (e.g., line scanners or planar projection imaging [16]) rather than just to computed tomography.

There are, of course, disadvantages of x-CSI compared with more traditional X-ray imaging systems, including the cost, system complexity, and pulse pileup [17]. Additionally, technical challenges remain which limit the utility of x-CSI in certain medical applications, primarily associated with counting losses and spectral distortions caused by pulse pileups at higher X-ray fluxes (10^8^–10^9^ photons mm^−2^ s^−1^). Developing x-CSI systems which can sustain high count rates (up to 10^9^ counts mm^−2^ s^−1^) without paralysing has been the focus of much work in recent years; however, these efforts often pay less attention to the issue of spectral distortion caused by the pulse pileup. Part of the reason may be the mistaken belief that pileup in x-CSI will manifest the same way as in other spectroscopic modalities, and so is already a well-understood phenomenon. Pulse pileup is a statistical phenomenon whereby an X-ray photon interacts with a pixel before it has finished processing a previous X-ray interaction. In gamma spectroscopy and related fields, this results in both a loss of counts (as the two photons are combined into a single event) and the recording of a photon with higher energy than either photon has in isolation.

This work is motivated to achieve two goals: (1) to test whether pileup-based spectral distortions manifest in the same way as in gamma spectroscopy and (2) to propose and test alternative count-triggering schemes designed to simultaneously allow higher count rates to be sustained and prevent pileup-induced spectral distortions.

As a result, this paper will highlight, for the first time, an additional form of spectral distortion that can be produced when multiple threshold–counter pairs are used, as is the case with x-CSI. It will show the surprising result that a negative number of photons can be calculated in a given energy bin given the right conditions, before arguing that such negative counts do actually occur regularly and are simply obscured by larger-scale effects. Finally, this discovery will be used to inform the design of new count-triggering schemes, with the aim of mitigating or eliminating the detrimental effects of negative counts. The results are alternative architectures which may not only improve x-CSI count rates, but also improve spectral fidelity at mid to high X-ray fluxes commonly used in clinical settings.

## 2. Materials and Methods

**Figure 2:**
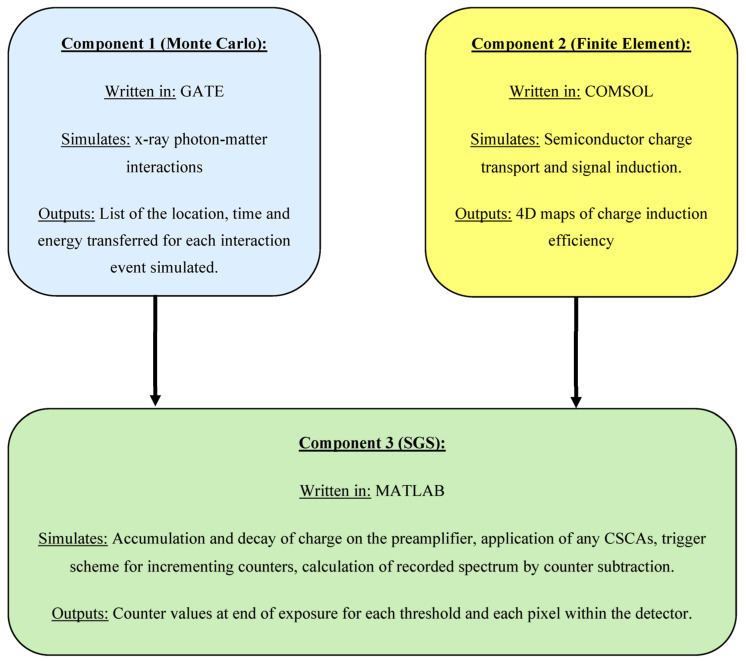
Schematic of the various components of CoGI, showing their name, simulation tools employed, goals, and outputs.

All simulations detailed in this work are performed using the previously validated simulation framework known as CoGI. CoGI was developed at the Institute of Cancer Research and the Royal Marsden NHS Foundation Trust by the Multimodality Molecular Imaging team for the purpose of modelling and optimising x-CSI detectors and is comprised of three primary components, as shown in Figure 2. Full details of this simulation framework can be found in previous publications [17,18], so only a brief overview will be given here.

### 2.1. Component 1: Monte Carlo Simultion

Component 1 in CoGI is a Monte Carlo (MC) model, run using the open-source GATE platform [19]. This simulation models the processes of X-ray generation and X-ray-matter interactions within the sensor material. The low-energy Livermoore physics library was implemented, including Rayleigh and Compton scattering, photoelectric absorption and X-ray fluorescence. The result is a list of the location, time, and energy transferred for each interaction between the X-ray beam and the sensor. The detector geometry, sensor material, and X-ray source information were taken from our previous work [17] and are summarised in Table 1. A monoenergetic irradiation was chosen so that the expected recorded spectrum would comprise sharp peaks (photopeak, escape peak and fluorescence peak), making spectral distortions easier to detect. 80 keV was chosen as it is high enough to allow the expected peaks to be well separated whilst still occurring at medically relevant energies. This energy is also relevant to other work ongoing in our group, including the previous simulation work we have done with CoGI to optimise other x-CSI system parameters [17,20].

### 2.2. Component 2: Finite Element Simulations

Component 2 in CoGI is a finite element model (FEM), performed using the commercial COMSOL [21] software (version 5.4). The FEM is used to calculate charge induction efficiency maps which allow the information regarding energy deposition location and time from GATE to be converted into electronic signals as a function of time. Material properties, pixel size, sensor thickness, and electric biasing were taken from our previous work [17] and are summarised in Table 2.

### 2.3. Component 3: Electronics and Data Processing Simulations

Component 3 in CoGI consists of a series of custom MATLAB codes, referred to collectively as SGS. These codes combine to model the electronics implemented on a given x-CSI application specific integrated circuit (ASIC). This component combines the outputs from GATE and COMSOL to reconstruct the charge produced on the preamplifier by each X-ray interaction. The charge integrated on the preamplifier was modelled as decaying exponentially according to the relation:

(1) $$C_{t}=C_{0}e^{-\lambda t}$$

where *C_t_* is the charge after time t, *C*_0_ is the full charge built on the preamplifier as a result of the photon interaction, t is the time after the peak charge is reached, and *λ* is the decay constant for the process. The decay constant was set such that the charge would decay to 1% of its original value in 100 ns by rearranging the equation to:

(2) $$\lambda =\frac{-\ln\left(\frac{C_{t}}{C_{0}}\right)}{t}$$

giving a decay constant of 4.61 × 10^7^. 100 ns is sufficiently long that pulse pileup events will not be uncommon at medically relevant fluxes (10^6^–10^9^ photons s^−1^ mm^−2^), whilst still being fast enough to be relevant to real-world applications based on ASICs currently available [22]. This produces a series of signal pulses with time for each pixel. CoGI allows for these signals to be altered based on a selection of “on-chip” charge-sharing correction algorithms (CSCAs), before the resulting signals are fed into a counter-triggering scheme which determines which counters, if any, are incremented as a result of the signal. In this work, the existing trigger scheme module was modified to allow newly proposed trigger schemes to be simulated in order to examine the effect of trigger scheme choice on perceived count rate and spectral distortion. For simplicity, no CSCAs were assumed in this work, so that any observed negative count rates could be assumed to be due to the choice of count-trigger scheme rather than an artifact of imperfect charge sharing correction. The different trigger schemes modelled are explained in Section 2.4. The common idea to all is that a series of thresholds are set, each with an associated counter which increments when the charge on the preamplifier is greater than that threshold.

Each trigger scheme was simulated as applied to 3, 5, 8, 24, and 130 energy thresholds. Existing x-CSI ASICs utilise a wide range of energy bin numbers and it would have been impractical due to time constraints to model all of them. The values chosen were thus to provide non-trivial representatives from the options already evaluated (3, 5, and 8), as well as to consider potential future systems which could have either a “broadly spectral” (24 bins each of 5 keV width) or a “fully spectral” (130 bins, each 1 keV width) design.

### 2.4. Counter Triggering Schemes Modelled

**Figure 3:**
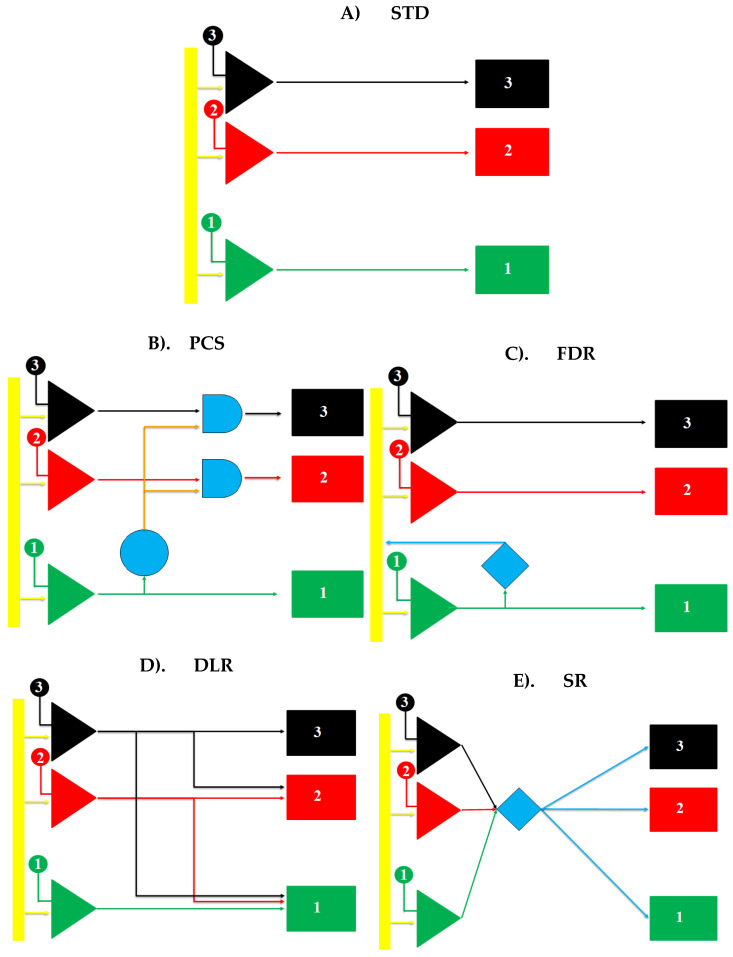
Schematics for the various count triggering schemes simulated in this work. Common symbols: Yellow rectangle (preamplifier), green/red/black numbered triangles (comparator to provide a threshold), green/red/black numbered rectangles (counter associated with same numbered/coloured comparator), straight lines (connections). Unique symbols: in (**B**), blue “D” shapes represent logical AND gates and the blue circle represents a device to provide an input of finite duration to the AND gates (e.g., a capacitor). In (**C**), the blue diamond represents a delay circuit which provides an offset, dt, between being triggered and resetting the anode (yellow rectangle). In (**E**), the blue diamond represents a second stage integraing circuit which counts the number of thresholds recently triggered (using an adjustable baseline) and then triggers that number of counters from lowest to highest.

The first trigger scheme modelled represents the standard scheme used in most PC/x-CSI ASICs (the “STD” scheme). The charge on the preamplifier is continuously compared against each threshold, with the associated counter being incremented only whenever the threshold is crossed from below: no change occurs to the counter when the charge crosses the threshold from above. In this configuration, each threshold–counter pair operates independently of the others. A visualisation of how this circuit operates can be seen in Figure 3A.

The first alternative trigger scheme we propose in this work is referred to as the “Premature Count Suppression” (PCS) scheme. In this approach, the lowest energy threshold (the “priming threshold”) serves as a controller for counting in all threshold–counter pairs. As this threshold is set just above the expected electronic noise floor of the system, it will only be able to trigger once sufficient charge from any previous interactions has bled off the preamplifier that the residual charge is within the range of expected electronic noise (and is thus negligible). This circuit could be physically constructed by placing logical AND gates between each threshold–counter pair, with the second input to the gate being from the prime threshold, as shown in Figure 3B. In the simulation, the AND gates remain open for 10 ns to allow for different rise times in the events.

The second alternative trigger scheme we propose in this work is referred to as the “Forced Delayed Reset” (FDR) scheme. In this approach, the priming threshold again serves to determine that a genuine photon has interacted with the system whilst the charge on the preamplifier is within the noise range of the system; however, here the priming threshold is the only one which requires modification. In this setup, when the priming threshold is triggered, it increments its counter, but also activates an additional circuit which, after a brief delay (100 ns here), shorts out the preamplifier, depleting its charge and resetting the circuit. The FDR trigger scheme is illustrated in Figure 3C. Two versions of this scheme were simulated here: one in which the reset was instantaneous, FDR(Inst), and one which had a finite deadtime of 10 ns after the 100 ns delay, FDR(DT).

The third alternative trigger scheme, we refer to here as the “Descending Line Responder” (DLR) scheme. This scheme operates by linking the output of each threshold to the input of both its counter and the counters of every threshold set lower than it. As a result, the triggering of a count in the *N*th bin will automatically increment all counters at *N* and below, meaning that negative counts become impossible. The DLR trigger scheme is illustrated in Figure 3D. Just like the STD scheme, this scheme runs in real time.

The final alternative trigger scheme we propose is referred to as the “Shift Register” (SR) scheme. This approach is the most complicated to physically implement, requiring an additional layer of processing between the thresholds and the counter. Instead of linking directly to an associated counter, each threshold servers as an input to a digital adder. The adder determines how many thresholds have been triggered, regardless of which ones they are, and then increments the same number of counters, starting from the bottom counter and working upwards in ascending order. This scheme carries with it the additional constraint that the spacing between all of the thresholds should be kept constant. The SR trigger scheme is illustrated in Figure 3E. In this simulation, the digital adder is assumed to work in real time.

The timing parameters used for signal shaping and processing are summarised in Table 3. A reminder of the various counting schemes and their acronyms can be found in Table 4.

## 3. Results

**Figure 4:**
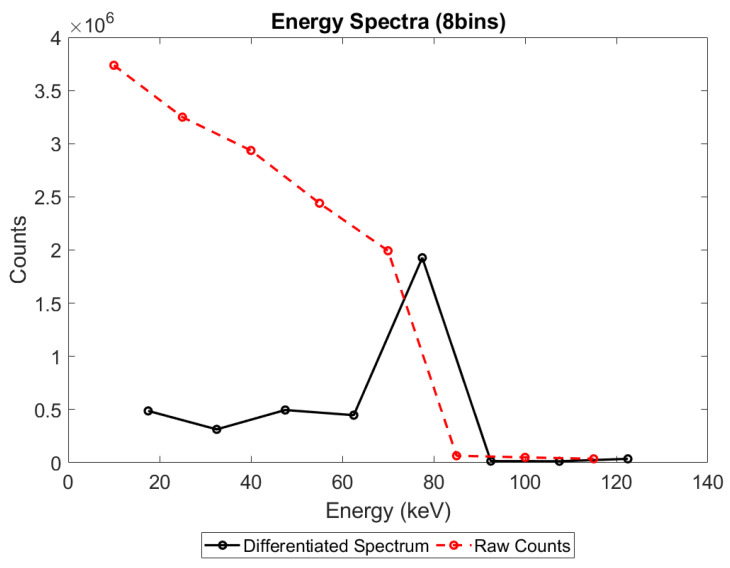
Raw counts from threshold counters (red dashed line) and the energy spectrum that is calculated as a result of counter subtraction (black solid line), as described in text. Data obtained from a simulated system with 8 energy bins, 10^8^ X-ray photons mm^−^^2^ s^−^^1^, 2 mm thick sensor, and 400 µm pixel pitch. 8 bins were chosen as this is the most used in a commercial x-CSI ASIC to date.

In all simulations, the number of events occurring within a given energy bin was determined by subtracting the number of counts on the counter at the upper edge of the bin from the counts on the counter at the lower edge of the bin. The resulting energy spectrum was then plotted by placing a marker at the mid-energy of each bin and connecting them with a straight line, as illustrated in Figure 4. This was done for each of the pixel pitches, sensor thicknesses, X-ray fluxes, and count-trigger schemes considered and used to calculate the count rate and spectral efficiency, as described in the Materials and Methods section.

**Figure 5:**
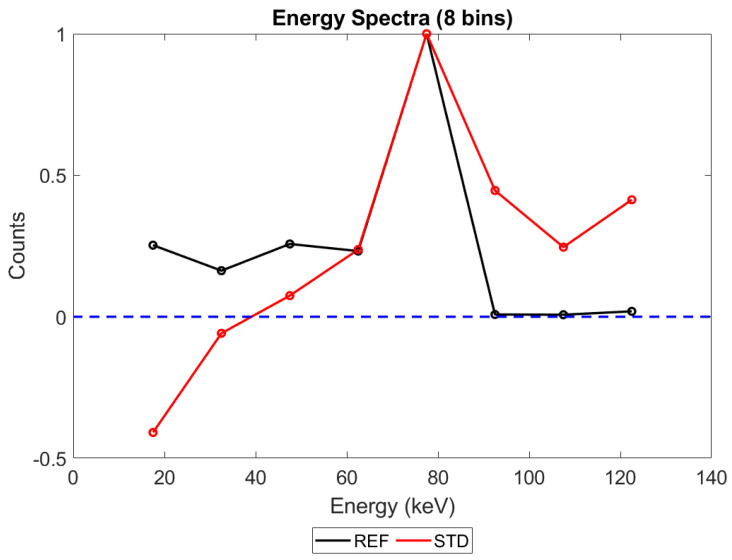
Comparison of the calculated energy spectrum produced by an ideal counting scheme (REF, black line) and the currently used counting scheme (STD). Data obtained from a simulated system with 8 energy bins, 10^8^ X-ray photons mm^−^^2^ s^−^^1^, 2 mm thick sensor, and 400 µm pixel pitch. Blue dashed line corresponds to zero counts and is added to allow negative counts to be more easily visualised.

Figure 5 shows the energy spectrum that would be produced by the counting scheme currently employed in x-CSI systems (STD, red line) at a relatively high but still medically relevant X-ray flux (10^8^ X-ray photons mm^−2^ s^−1^, 2 mm thick sensor, and 400 µm pixel pitch). Though the raw number of counts on each counter are positive, the subtraction process used to generate an energy spectrum causes the number of photons in bin 1 to turn negative. This phenomenon, which we refer to here as “negative counting”, will be explored in more detail in the discussion section. It suffices to say for now that the alternative counting schemes we propose are designed to limit this artefact and its associated spectral distortions. For comparison, idealised reference data is provided (REF, black line). This data was produced by processing the simulation data using an idealised counting scheme, in which events were grouped into 1 ns bundles and then the signal from each bundle was calculated in isolation, assuming zero background charge. This approach should severely limit the contribution of pulse pileup and completely remove the possibility of negative counts from the idealised reference (though it is still possible for true coincidence photons within a pixel to increase the registered energy).

**Figure 6:**
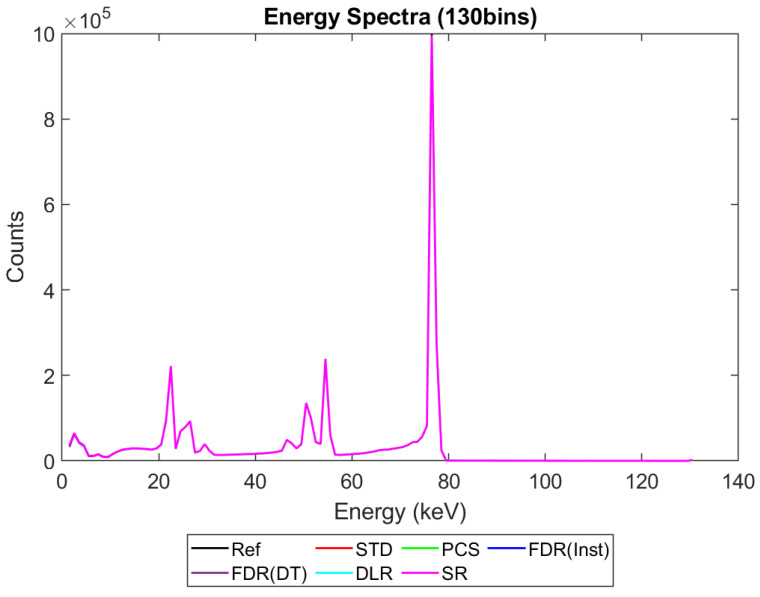
Energy spectrum obtained from a system with a 1.5 mm sensor thickness, a 250 µm pixel pitch, and 130 energy bins. The X-ray flux simulated was 10^6^ photons mm^−^^2^ s^−^^1^. All spectra are plotted; however, they perform almost identically at this low flux, leading only the last line plotted visible over the top of the others.

**Figure 7:**
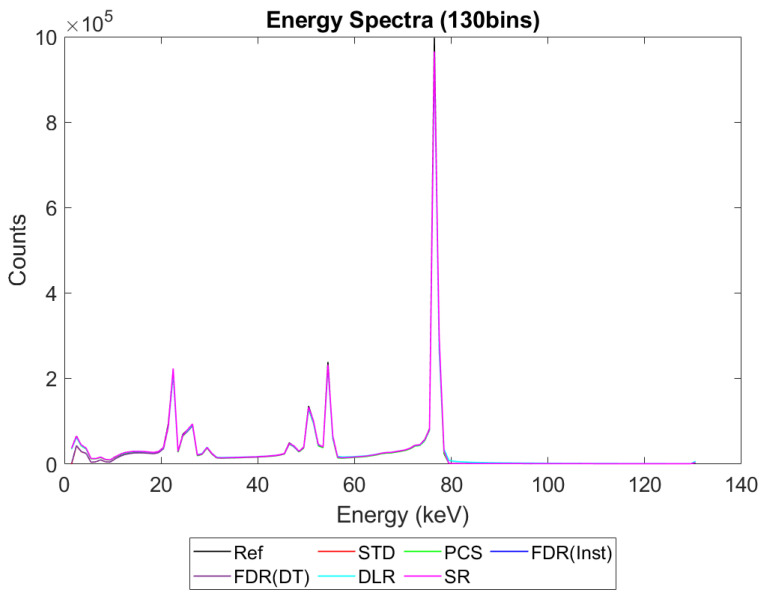
Energy spectrum obtained from a system with a 1.5 mm sensor thickness, a 250 µm pixel pitch, and 130 energy bins. The X-ray flux simulated was 10^7^ photons mm^−^^2^ s^−^^1^. All spectra are plotted; however, they perform equally well at most energies at this flux, with only slight differences visible at the lowest and highest energies (where FDR and DLR respectively are visibly different).

**Figure 8:**
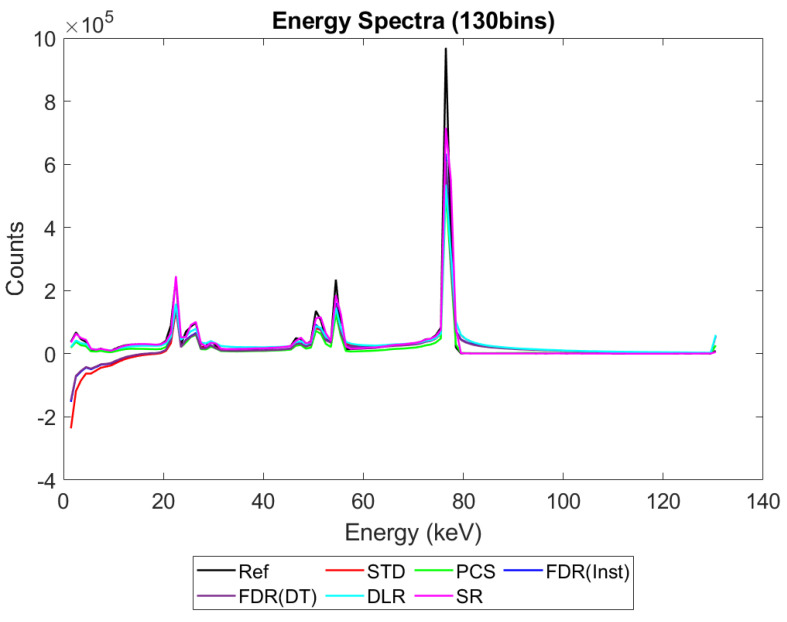
Energy spectrum obtained from a system with a 1.5 mm sensor thickness, a 250 µm pixel pitch, and 130 energy bins. The X-ray flux simulated was 10^8^ photons mm^−^^2^ s^−^^1^.

**Figure 9:**
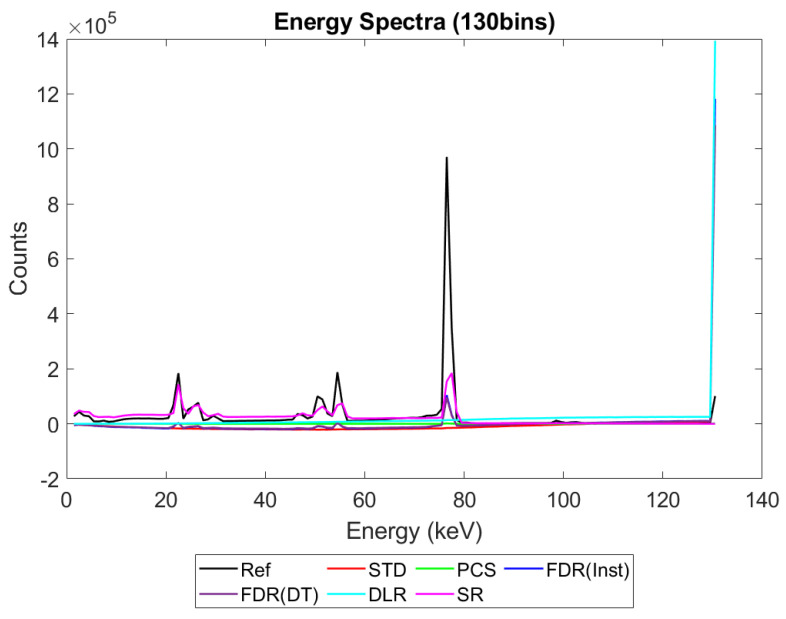
Energy spectrum obtained from a system with a 1.5 mm sensor thickness, a 250 µm pixel pitch, and 130 energy bins. The X-ray flux simulated was 10^9^ photons mm^−^^2^ s^−^^1^.

**Figure 10:**
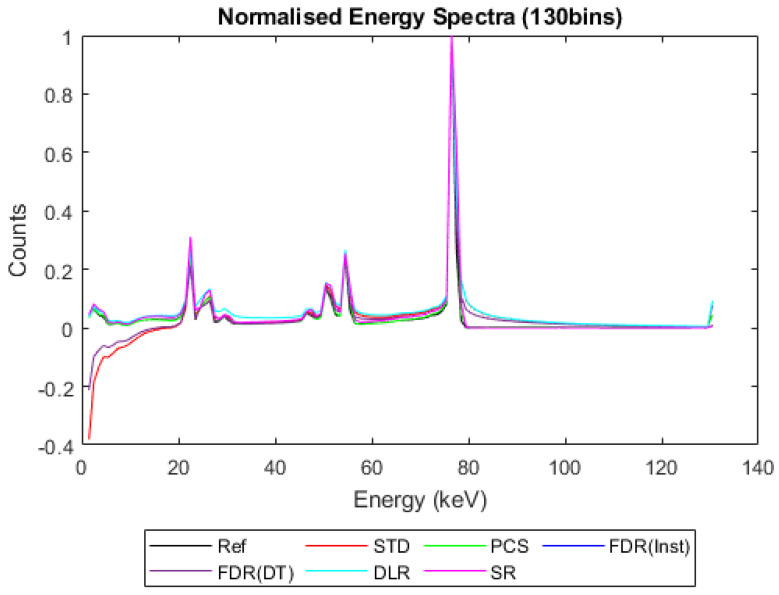
Normalised energy spectrum obtained from a system with a 1.5 mm sensor thickness, a 250 µm pixel pitch, and 130 energy bins. The X-ray flux simulated was 10^8^ photons mm^−^^2^ s^−^^1^.

**Figure 11:**
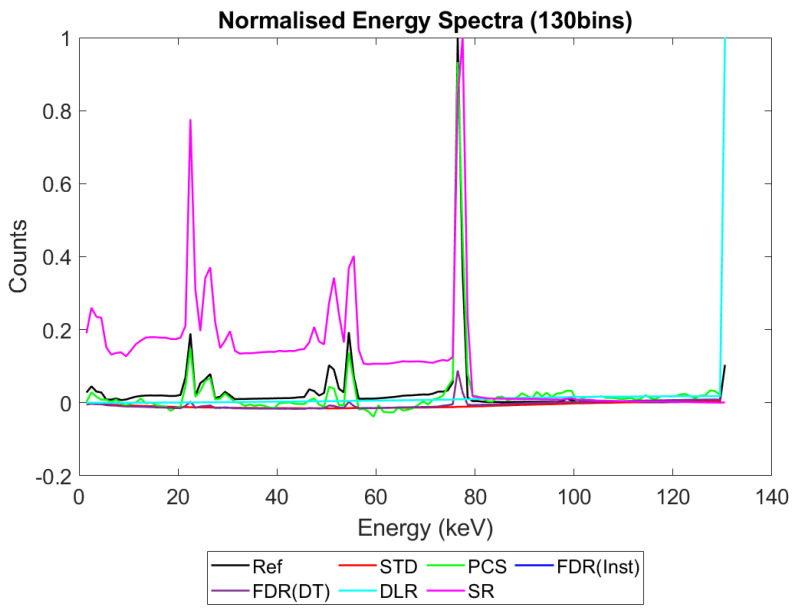
Normalised energy spectrum obtained from a system with a 1.5 mm sensor thickness, a 250 µm pixel pitch, and 130 energy bins. The X-ray flux simulated was 10^9^ photons mm^−^^2^ s^−^^1^.

**Figure 12:**
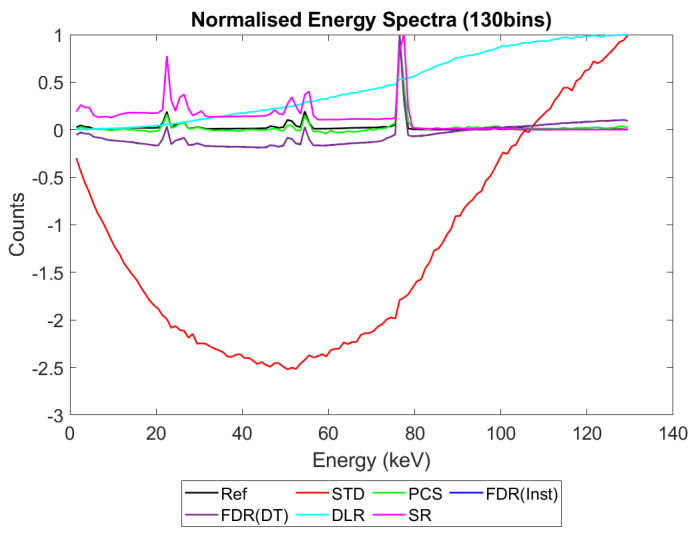
Energy spectrum normalised after removing data from the last energy bin, obtained from a system with a 1.5 mm sensor thickness, a 250 µm pixel pitch, and 130 energy bins. The X-ray flux simulated was 10^9^ photons mm^−^^2^ s^−^^1^.

Figure 6, Figure 7, Figure 8 and Figure 9 show the performance of the various alternative trigger schemes proposed at X-ray fluxes of 10^6^ (Figure 6) to 10^9^ (Figure 9) photons mm^−2^ s^−1^. The underlying simulations used the highest spectral resolution (130 energy bins), so that the spectral performance of each can be visualised the most clearly. The results shown are for illustrative purposes and assume a 1.5 mm thick sensor with a 250 µm pixel pitch, as these values were determined by us to be optimal for imaging a monoenergetic source at this energy in previous work [17,20]. The use of both raw and normalised energy spectra is helpful as each emphasises different aspects of the various triggering schemes: raw plots are better for relaying the counting efficiency of each scheme (as the areas under the curve represent the total number of counts) whilst plots normalised to maximum peak height are better for assessing spectral efficiency (by allowing the relative heights of each bin to be more easily compared). For this reason, normalised energy spectra for 10^8^ and 10^9^ photons mm^−2^ s^−1^ were produced (Figure 10 and Figure 11 respectively). DLR and STD schemes assign almost all events to the highest energy bins at the highest fluxes, so a second normalised plot is produced for this X-ray flux (Figure 12), where the events in the final energy bin are removed before normalisation. Normalised versions of Figure 6 and Figure 7 are not provided as they did not differ in any meaningful way from their unnormalized counterparts.

It is clear from Figure 6, Figure 7, Figure 8, Figure 9, Figure 10 and Figure 11 that whilst at low fluxes (where pileup is almost non-existent) all trigger schemes perform approximately equally well, at high fluxes (where pileup dominates) the various trigger schemes begin to differ markedly, with some maintaining spectral performance whilst losing counts (e.g., PCS) whilst others maintain counts but at the expense of spectral performance (e.g., DLR).

Whilst much qualitative information can be gained from analysing such figures, an inordinate number of them would be required to account for every possible combination of variables simulated. Consequently, a reduction in the dimension of the data was required. This was performed by calculating for each energy spectrum two values: the spectral efficiency and the counts recorded.

The spectral efficiency is defined as:

(3) $$S_{eff}=\frac{N_{P}}{N_{T}}$$

where *S_eff_* is the spectral efficiency, *N_P_* is the number of counts recorded in the photopeak, and *N_T_* is the total number of counts recorded by the detector. Note that, to avoid the potential of divisions by zero or negative efficiencies, bins with negative counts were treated as having zero counts for the purposes of calculating this metric. Due to the binned nature of the dataset, *N_P_* was assumed to be equal to the number of counts in the energy bin(s) which contained the photopeak, as identified in Table 5. Representative 130 energy bin spectra were used to determine the range of energies spanned by the photopeak for the purposes of determining which energy bins it fell into.

The total number of counts recorded across all bins, *N_T_*, was used as a measure of counts recorded; however, as before, negative counts were treated as 0 for the purpose of this analysis.

**Figure 13:**
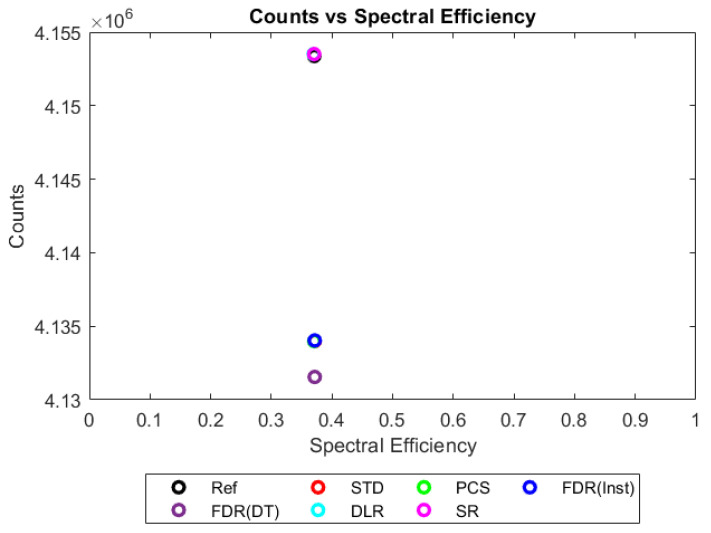
Plot showing the spectral efficiency and counting performance of the various count-triggering schemes considered on a system with pixel pitch 250 µm, sensor thickness 1.5 mm, and 130 energy bins, at an X-ray flux of 10^6^ photons mm^−^^2^ s^−^^1^. The y-axis has been zoomed in to allow schemes to be more clearly differentiated as comprising 3 groups: top group (REF, DLR and SR), middle group (STD, FDR(Inst) and PCS), and bottom group (FDR(DT)).

**Figure 14:**
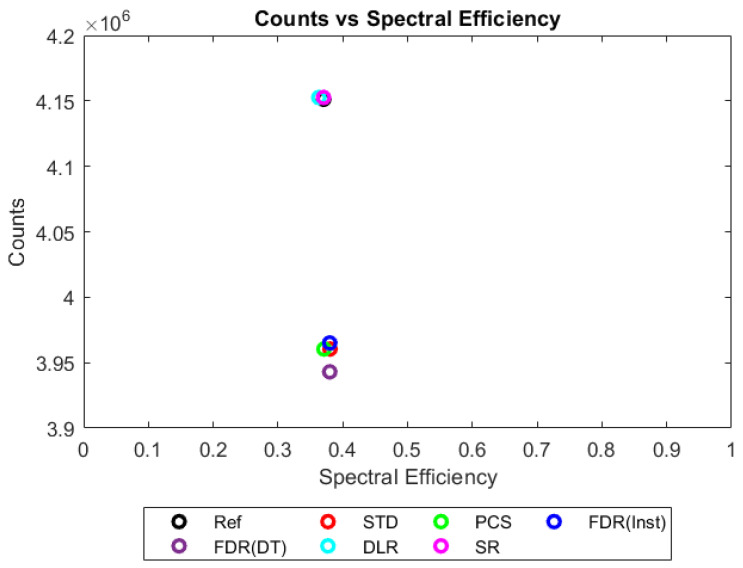
Plot showing the spectral efficiency and counting performance of the various count-triggering schemes considered on a system with pixel pitch 250 µm, sensor thickness 1.5 mm, and 130 energy bins, at an X-ray flux of 10^7^ photons mm^−^^2^ s^−^^1^. The y-axis has been zoomed in to allow schemes to be more clearly differentiated as comprising 3 groups: top group (REF, DLR and SR), middle group (STD, FDR(Inst) and PCS), and bottom group (FDR(DT)).

**Figure 15:**
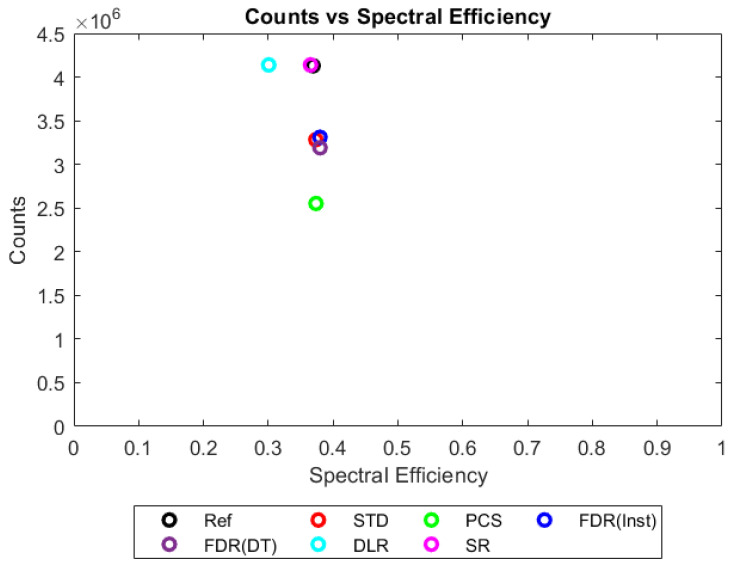
Plot showing the spectral efficiency and counting performance of the various count-triggering schemes considered on a system with pixel pitch 250 µm, sensor thickness 1.5 mm, and 130 energy bins, at an X-ray flux of 10^8^ photons mm^−^^2^ s^−^^1^.

**Figure 16:**
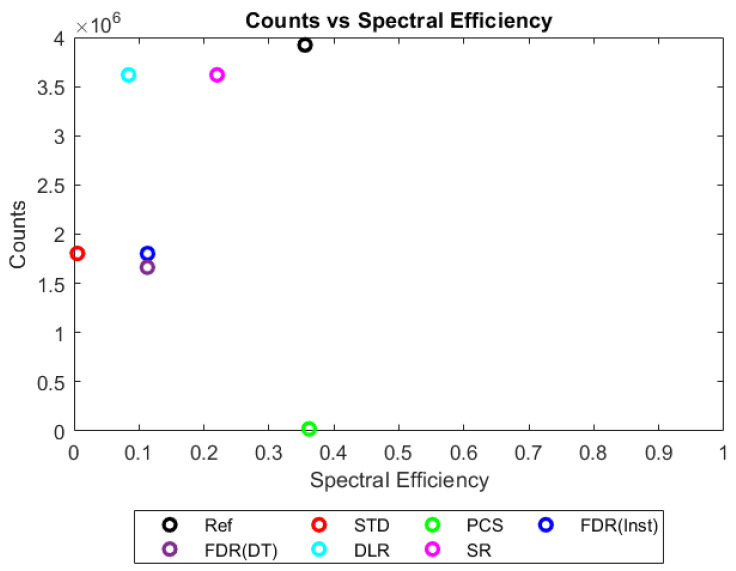
Plot showing the spectral efficiency and counting performance of the various count-triggering schemes considered on a system with pixel pitch 250 µm, sensor thickness 1.5 mm, and 130 energy bins, at an X-ray flux of 10^9^ photons mm^−^^2^ s^−^^1^.

Values for each metric could then be plotted in 2D to compare a trigger scheme’s spectral efficiency with its counting efficiency at a range of X-ray fluxes, as shown in Figure 13, Figure 14, Figure 15 and Figure 16. The plots shown consider only a single combination of sensor thickness, pixel pitch, and number of energy bins (2 mm, 250 µm and 130 bins); however, they offer a good illustration of how the performance of the various schemes changes with increasing pulse pileup, here produced by increasing the X-ray flux from 10^6^ to 10^9^ photons mm^−2^ s^−1^. Similar plots were made for all possible combinations of pixel pitch, sensor thickness, number of energy bins, and X-ray fluxes. The distance in these 2D plots between a given trigger scheme and the idealised reference was used as a performance metric for that trigger scheme. Axes were scaled such that the idealised reference data had coordinates of (1,1), so that an equal fractional change in either spectral efficiency of counting efficiency would produce an equal change in distance. Distance was defined as:

(4) $$D_{tsr}=\sqrt{{\left|1-\frac{x_{ts}}{x_{rs}}\right|}^{2}+{\left|1-\frac{y_{ts}}{y_{rs}}\right|}^{2}}$$

where *D_tsr_* is the distance between the points for the idealised reference system and the trigger scheme investigated, *x_ts_* and *x_r_* are the spectral efficiencies of the trigger scheme and reference point, respectively, and similarly, *y_ts_* and *y_r_* are counts recorded for the trigger scheme and reference pointm respectively. This produces a metric, *D_tsr_*, which ranges from 0 to √2, with 0 meaning perfect agreement with the idealised system and √2 representing a complete failure of counting and spectral efficiencies.

**Figure 17:**
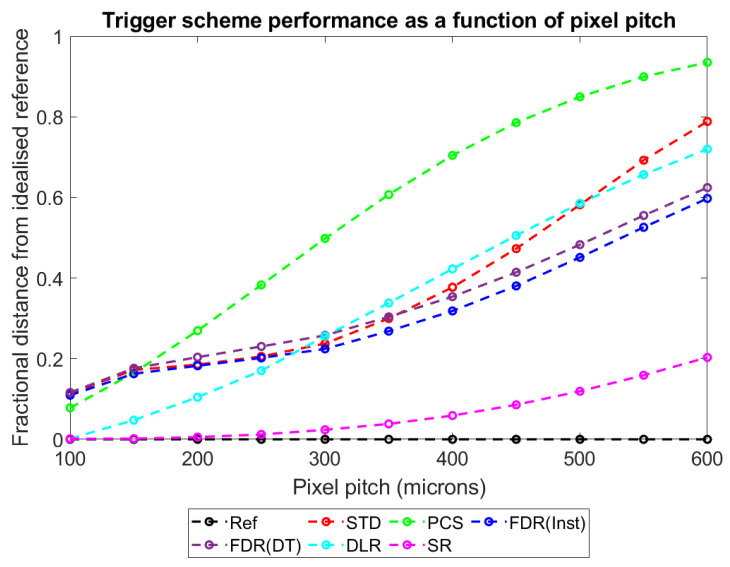
Plot of count scheme performance as a function of pixel pitch. Other system parameters were: sensor thickness 1.5 mm, 130 energy bins, and an X-ray flux of 10^8^ photons mm^−^^2^ s^−^^1^.

**Figure 18:**
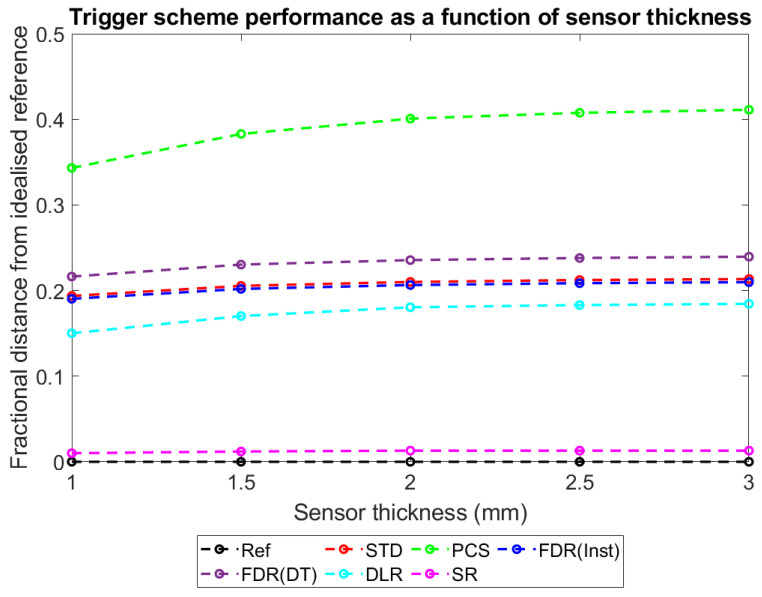
Plot of count scheme performance as a function of sensor thickness. Other system parameters were: pixel pitch 250 µm, 130 energy bins, and an X-ray flux of 10^8^ photons mm^−^^2^ s^−^^1^.

**Figure 19:**
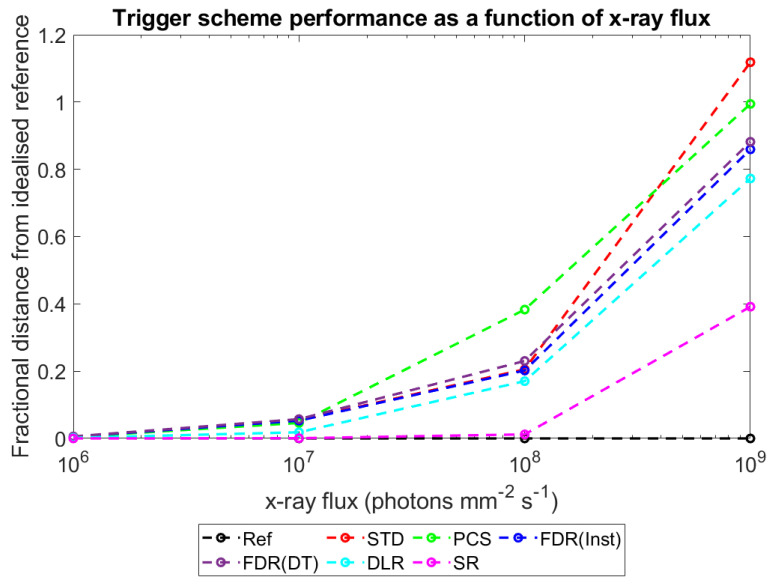
Plot of count scheme performance as a function of X-ray flux. Other system parameters were: sensor thickness 1.5 mm, pixel pitch of 250 µm, and 130 energy bins.

**Figure 20:**
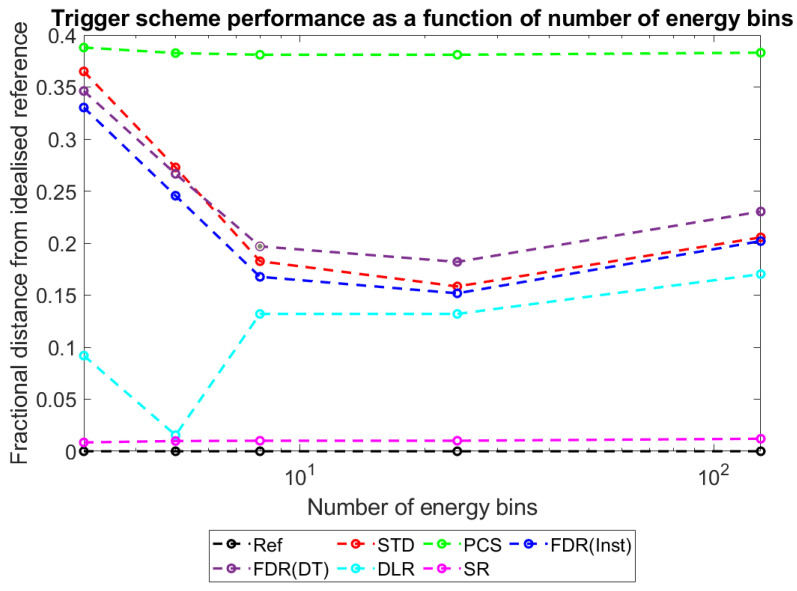
Plot of count scheme performance as a function of number of energy bins. Other system parameters were: sensor thickness 1.5 mm, 250 µm pixel pitch, and X-ray flux 10^8^ photons mm^−^^2^ s^−^^1^.

With this metric established and explained, plots were produced to show how D_ts-r_ varies for each of the different trigger schemes as a function of the major simulated variables: pixel pitch (Figure 17), sensor thickness (Figure 18), X-ray flux (Figure 19), and number of energy bins (Figure 20). These plots will be explored further in the Discussion section.

## 4. Discussion

The results presented are intended to demonstrate, for the first time in the literature, two things:

(1) that negative counts exist in traditional photon counting systems at medically relevant fluxes; and
(2) how a range of alternative count-triggering schemes designed to mitigate these negative counts can affect overall system performance.

This paper is not designed to determine how geometric considerations impact on charge sharing, detection probability, or counting accuracy. For a more detailed discussion on how geometric parameters (e.g., pixel pitches and thicknesses) affect the performance of x-CSI detectors, see our previous work [17].

### 4.1. Negative Counts: Mechanism and Evidence from Simulations

**Figure 21:**
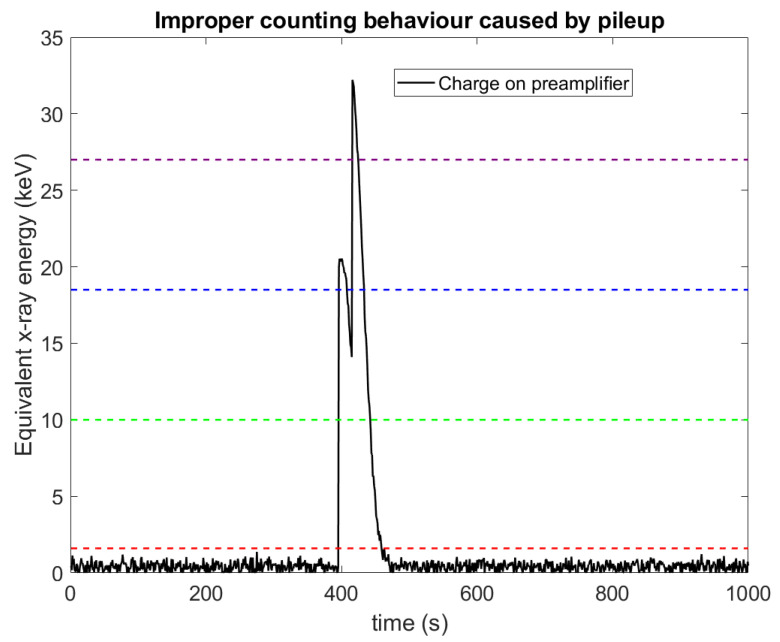
Plot showing how pulse pileup can cause negative counts to register in a system. The first photon (lower peak starting around 400 ns) causes the lowest 3 counters (red, green, and blue thresholds) to increment. The second photon (higher peak starting around 420 ns) arrives after the charge falls below the blue threshold, but is still above the red and green thresholds. As a result, the second event triggers the 3rd and 4th counters (blue and purple thresholds) but not the lower 2 (red and green). How this leads to a negative count is further explained in Table 6.

The traditional x-CSI count-triggering scheme is illustrated in Figure 1 and explained more fully in Figure 3A works perfectly well for single X-ray photon detections; however, the situation is more complicated where multiple photons interact with a pixel within a very short space of time, as shown in Figure 21. The process of collecting the charge produced by an X-ray interaction within the sensor, integrating the charge on a preamplifier, and then discharging this charge to reset the circuit takes a finite amount of time, sometimes up to a few hundred nanoseconds (a 100 ns exponential decay was used in this work). If a second X-ray photon should interact with the sensor during this interval, the charge from the second photon will be summed with the remaining charge in the circuit, resulting in a larger signal than would otherwise be the case. This phenomenon is known as pulse pileup. Pulse pileup is well understood in gamma spectroscopy applications where it is considered to have two primary effects: lost counts (affecting activity calculations) and the formation of additional peaks (“pileup peaks”). The different implementation of x-CSI circuits allows for a new, more subtle spectral distortion effect however, which we refer to here as “negative counts”.

A negative count is not the same as a lost count and is arguably unlike any of the pileup effects commonly discussed. Lost counts and pileup peaks both represent an error that affects the data recorded during the pileup process: 2 photons may be recorded as 1, or the second photon may be shifted to a higher energy, but the rest of the data acquired outside of this pileup is unaffected. This is not the case with negative counts: negative counts can degrade spectral data acquired before or after the pileup event in question, due to the way that spectral information is encoded and extracted in x-CSI. As explained in Figure 4, the number of photons detected in an energy bin is determined by subtracting the counts on the upper threshold counter for the bin from the lower threshold counter. This process assumes that an event which rises above threshold *N*+1 must also rise above threshold *N*. This assumption may be flawed in pileup situations, such as that shown in Figure 21 which shows the case of two 20 keV photons arriving within a short time of each other. In the time interval between the two photons arriving, the preamplifier charge produced by the first photon is able to decline below threshold 3 (blue dashed line), but not below thresholds 1 or 2. The counts produced on each threshold counter, as well as the resulting counts calculated for the associated energy bins, are shown in Table 6.

We are thus left with the interesting result that these two photons have been recorded in 3 energy bins, but the total addition to system counts is 1 (−1 + 1 + 1). In one sense, this pileup behaviour is familiar, with two photons being summed together to produce a single additional count in the system at an energy approximately equal to their sum (1 count added to the >27 keV bin, and a total of 1 additional count in the system overall). Unexpectedly, however, by subtracting 1 from the 10–18.5 keV bin and adding 1 to the 18.5–27 keV bin, this pileup has also had the effect of shifting the score of another photon not involved in the pileup. Indeed, if the 10–18.5 keV bin is currently empty then perhaps the photon that will be shifted has not even arrived yet. If it never arrives, the result will be a negative number of counts in this energy bin, as demonstrated in Figure 5 which models a not-unrealistic clinical system (10^8^ X-ray photons mm^−2^ s^−1^, 2 mm thick sensor, and 400 µm pixel pitch). Obviously, negative numbers of photons are not physically possible; rather, these results illustrate the potential issues that can arise if x-CSI systems are operated at X-ray fluxes in which the assumption that X-ray interactions are independent is invalid.

Now is a good time to address the immediately obvious objection: why are negative counts not routinely seen in physical systems? To be clear, it is highly unlikely that anyone would see negative counts on a physical system at the moment due to a combination of four factors:

(1) Negative counts occur only within a specific range of inter-photon timings: too short a time between photons will cause the events to pile up entirely (only one event will be counted, at an energy equal to the sum of the two photons) whilst too long a time between them will leave residual charge less than the lowest threshold, resulting in all thresholds again being incremented (this is how an x-CSI system would ideally work).
(2) The simulated radiation source was monoenergetic; however, most practical x-CSI setups employ a polychromatic beam. As both high- and low-energy photons trigger low threshold counters, but only high-energy photons trigger high threshold counters, low-energy thresholds will typically produce more counts than higher-energy ones (neglecting pileup for now). This means that even if every high-energy photon caused a lost count in the low-energy bins, the counts in the lower bins are so high by comparison that the overall count will still not turn negative. Instead, the ratio of low to higher energy photons would be shifted in a way confusable with beam hardening.
(3) X-ray tubes produce spectra with fewer photons of the highest energy and more in the low- to mid-energy range. As with the preceding point, this leads to more counts on the lower threshold counters to begin with and masks the effect of the negative counts.
(4) In order to fall below the threshold *N* during the time interval between photons, *dt*, the charge produced by a photon needs to lie within some specific range of charges, *dC*, above *N*. As energy bins get wider, the proportion of photons within the bin which lie within the range *dC* will decrease, making the bin shifting that produces negative counts less likely. As most modern x-CSI systems use a small number of thresholds, it could be assumed to a first approximation that the majority of pileup events do not involve charges dropping below a threshold, masking the effect of these negative counts. It thus needs to be remembered that though a large number of energy bins may appear to show a detailed energy spectrum, the x-CSI counting circuitry means that additional thresholds do not necessarily correlate with better quality spectral data, as the higher spectral resolution may be traded off against increased spectral distortion, at least for fluxes at which pileup is significant.

**Figure 22:**
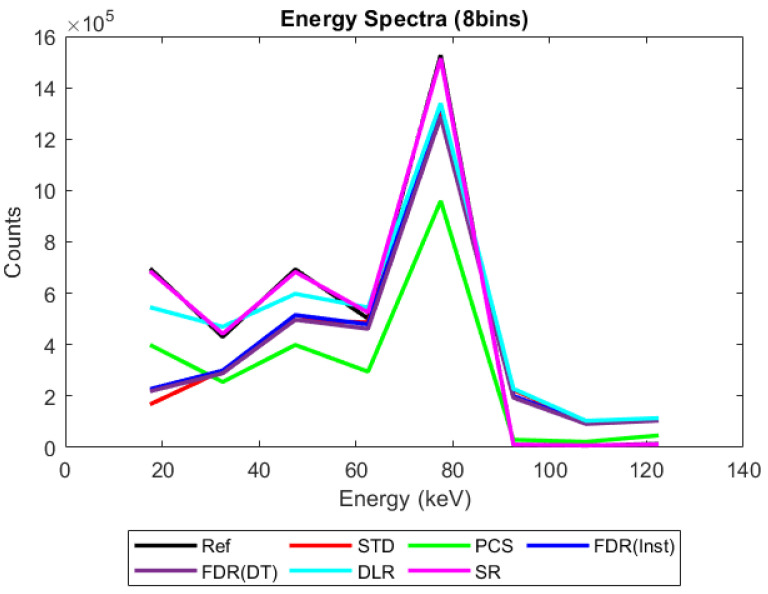
Data used to produce Figure 8 (sensor thickness 1.5 mm, pixel pitch 250 µm, and X-ray flux 10^8^ photons mm^−^^2^ s^−^^1^) processed with 8 energy thresholds instead of 130. Note that the negative counts seen when 130 thresholds are used now appear simply as lower counts in the lowest of 8 energy bins. The REF data set lies almost exactly behind the SR data set.

That negative counting effects become more apparent at higher numbers of bins is a particularly important point if the field moves toward extracting more spectral data from an acquisition by increasing the number of energy thresholds used, and therefore warrants additional evidence. To support this claim, then, compare Figure 8 with Figure 22 below. Figure 22 represents the exact same physics simulated as Figure 8, but with the output processed with 8 energy bins instead of 130. What appears as negative counts in a 130-bin system instead manifests as a reduction of counts in the associated bins of the 8-bin system. Unequivocally demonstrating the importance of understanding this phenomenon, notice that the result of these pileups is the loss of an entire spectral peak at this resolution: the reference data descends from a peak at 20 keV to a valley at 30 keV before rising to a second peak at 40 keV, whilst the STD trigger scheme makes no descent between 20 and 40 keV, instead rising more gradually and providing the illusion that the peak at 40 keV is both the lowest energy peak and much broader than it actually is. Considering the physics behind this irradiation, what we are seeing here is both a loss of the X-ray fluorescence peaks associated with the CdTe and an underestimation of the energy resolution with which the escape peaks can be resolved.

It should thus be apparent to the reader that, even though negative counts may not be seen in a system, they can still constitute a significant source of spectral distortion.

### 4.2. Performance of Alternative Count-triggering Schemes: Energy Spectra

When considering the performance of the various trigger schemes proposed in this section, it is important to remember they are compared with the idealised reference system. Additionally, whilst full spectra are only shown here for the case of 130 energy bins, negative counts can manifest for any number of energy bins, as shown in Figure 5 for 5 energy bins. 130 bins was simply chosen as the increased level of detail makes the losses in spectral fidelity more visually evident. Finally, it should be noted that where significant overlap occurs between different trigger schemes, especially at the lowest X-ray fluxes, their spectra may be difficult or impossible to separate by eye. Figure captions describe the location of the STD and reference schemes for each figure, and the text in this section will explain relevant trends to the reader where this overlap makes interpretation more difficult.

Figure 6, Figure 7, Figure 8 and Figure 9 show the X-ray spectra recorded by the various trigger schemes at a range of clinically relevant X-ray fluxes (10^6^–10^9^ photons mm^−2^ s^−1^) compared with the spectrum that the reference system would produce. At very low to mid fluxes (10^6^–10^7^ photons mm^−2^ s^−1^), all trigger schemes appear to perform equally well: so similarly, in fact, that they cannot be distinguished in Figure 6 at all. Slight differences between the count-triggering schemes can be seen at the lowest and highest energies in Figure 7, where the trigger schemes are split into two groups: SR, DLR, and PCS match the reference data more closely than STD and both versions of FDR, which all experience a slight reduction in counts at this point, an indication that pulse pileup is beginning to creep into the simulation and cause reductions to the counts in the lowest energy bins as described in Section 4.1. DLR also produces a very slight over-count at the highest energies, but broadly speaking there is little difference between the various trigger schemes at these fluxes. This is reassuring as it implies that the proposed trigger schemes do not suppress negative counts at the expense of their performance in low flux regimes.

Figure 8 shows the performance as the flux increases to the levels more common of medical CT (10^8^ photons mm^−2^ s^−1^). The idealised reference spectrum is not visible in this figure as it is overlapped almost perfectly by the SR spectrum. At this flux, the effects of negative counting become noticeable at the lower end of the X-ray spectrum, with STD and both FDR schemes producing negative counts. These schemes also exhibit more significant high-energy tailing after photopeaks (most visible on the 80 keV photopeak) and a significant uptick in counts in the final energy bin. This behaviour is also evident in the DLR scheme, though it is better at avoiding negative counts at lower energies. In contrast, both PCS and SR continue to match the reference spectrum well at this X-ray flux, without either negative counts or additional high-energy tailing (again most evident after the 80 keV photopeak where they return to zero counts at about the same speed as the reference spectrum). Using the normalised version of this data set to better see the spectral fidelity of each approach (Figure 10), we can see that the PCS scheme fits the reference data most closely, except in the last energy bin where it makes a marked uptick. The SR scheme is a close second, though it overestimates the relative height of the escape and fluorescence peaks slightly and generally produces a poorer energy resolution than PCS or the reference system, as measured by the FWHM of the primary photopeak (though it does not suffer from the uptick of counts in the highest energy bin that all of the other schemes do).

Figure 9 shows how the counting schemes perform at the highest medical X-ray fluxes (10^9^ photons mm^−2^ s^−1^). Very interestingly, the STD scheme can still be seen to suffer from negative counts; however, rather than a sharp drop in counts at the lowest energies, the lost counts are now spread over a much wider range of energies, with negative counts visible between 0 and 80 keV. This indicates that increasing the pileup increases the range of energies over which the lost counts are spread, resulting in an apparently subtler effect when the spectrum is observed overall. Such a shallow, broad range of negative counts produced by high-energy photons could be easily obscured by the polychromatic nature of an X-ray tube, especially considering the preponderance of low-energy photons within the tube spectrum, which may again go some way toward explaining why these effects are not often observed in physical experimental systems. The majority of counts at this flux are concentrated in the last energy bin (the STD scheme rises sharply in the last energy bin but is hidden by the DLR scheme in the figure). The reader may rightly ask whether the negative counts now purely cause a large increase in the final energy bin, and so whether removing the final bin before normalising might restore some of the “lost” spectral information. To reassure such readers that the problem of negative counts is more fundamental than that, Figure 12 was produced. This figure shows that STD and DLR schemes do not retain accurate spectral information simply obscured by the highest energy bin, but rather that the same processes which cause counts to accumulate in the highest energy bin have also destroyed the spectral content of the lower energy bins. In the case of the STD scheme, this can clearly be seen to result in a significant number of negative counts for almost the entirety of the real energy spectrum (<80 keV).

The simulations here used a single value for the shaping time of the system, whereas shifting this value may result in negative counts becoming dominant at a different X-ray flux, though at the risk of increasing pulse pileup or ballistic deficit. Nevertheless, the general results presented here demonstrate both the existence of negative counts and their potential degrading effects on energy spectra, and as the simulation parameters were chosen to be relevant to existing x-CSI systems, the existence of this effect is important.

Returning to Figure 9 and considering the alternative trigger counting schemes proposed in this work, PCS and DLR both appear to significantly suffer because of pulse pileup. The poor performance in the PCS scheme is primarily characterised by a paralysis which results in almost no counts being registered, whilst the DLR scheme sees a spectral shift, with almost all counts assigned to the highest energy bin, as already noted. Despite the higher number of overall counts in the DLR approach however, normalised data shown in Figure 11 and Figure 12 reveals that PCS preserves the spectral information extremely well, with the closest fit to the reference data in the 0–80 keV range. Surprisingly, this scheme still exhibits negative counts at several energies, despite it specifically being designed to avoid this. The best explanation we can currently offer for this phenomenon is that the simulated AND gates are not instantaneous in operation, with a temporal resolution of 10 ns. It is thus possible that, at a sufficiently high flux, the process of a charge falling below threshold and rising again to retrigger the same threshold (and thus causing a negative count) could happen within the interval of 10 ns. Unfortunately, the current implementation of CoGI does not allow us to investigate this issue further. Should this issue be of interest to any readers, a new simulation could be produced to test it.

Figure 9, Figure 11 and Figure 12 also show that the FDR schemes combine the performance of STD and PCS schemes. FDR schemes exhibit a large rise in counts in the last energy bin (obscured by the DLR data) and broadly track the base count rate of the STD system, including the broad negative count regions. Though negative overall, the FDR schemes still retains an appreciable level of spectral features and a modest count rate, with spectral features visible in the raw spectra without normalisation.

The final scheme to comment on is the SR trigger scheme, and its performance warrants a more detailed discussion and explanation. From Figure 9 it is evident that this count-triggering scheme maintains an impressive amount of both spectral detail and counting speed, even at the highest medically relevant X-ray fluxes considered in this work (10^9^ photons mm^−2^ s^−1^). Indeed, the count numbers are close to the idealised reference spectrum in almost all energy bins, with all of the major spectral peaks still present. The differences between this trigger scheme and the idealised reference can be divided into two observations: the ratio of peak heights is not preserved (with higher energy peaks progressively being undersized by comparison) and the SR scheme does not produce an uptick in counts in the highest energy bin. It initially is tempting to speculate that the second of these points implies that the SR scheme may actually be able better at avoiding pulse pileup effects than the idealised reference. Given the novel design of this scheme, however, and the previously mentioned overestimation of lower energy peaks compared with higher energy ones, it seems more likely that these differences are a result of pileup manifesting differently in this system than in the reference case. To flesh out the differences in pileup processing, consider how a true coincidence of two 80 keV photons would be processed under each count-triggering scheme. In the case of the idealised reference, the energy of the two photons would be summed and then compared with all thresholds. As 160 keV is greater than all thresholds, they would all be incremented, and after binning, this would leave a single count in the highest energy bin, explaining the uptick seen in the reference case. In the SR scheme, such a pileup would also trigger all available thresholds; however, crucially, unlike the reference case, the SR scheme often experiences residual charge from a previous event as it does not clear this charge instantly. Consequently, whilst true coincidence counts manifest in the reference scheme as counts in the top energy bin, the same phenomenon could lead to counts at a wide range of energies in the SR system. The exact energy at which the pileup appears under the SR scheme will depend on the level of residual charge in the circuit at the time of the interaction, with higher levels of residual charge leading to the coincidences registering at lower energies (due to fewer thresholds being available to trigger). Unfortunately, it is not easy to investigate this claim directly with the simulations performed by determining where true coincidence counts end up for each count-triggering scheme. Nevertheless, from Figure 9 and Figure 11 we can see that the SR scheme overestimates counts at lower energies, which is consistent with the explanation offered here. Of final note is that the SR scheme drops sharply to zero after the primary photopeak at 80 keV at all of the X-ray fluxes studied. This is in contrast with the DLR and both FDR schemes which exhibit high-energy tailing after the photopeak which increases in extent with the increasing X-ray flux. As these energies could only be accessed by pileup in this monoenergetic setup, it is a good indication that the SR scheme does not produce artificially high-energy counts as a result of pulse pileup. The fact that photons can produce counts at the correct energy bin or lower but not in higher energy bins is in contrast to all of the other counting schemes considered, and could potentially improve performance in spectral decomposition tasks by reducing the spectral overlap between bins.

### 4.3. Performance of Alternative Count-Triggering Schemes: Count Rate vs. Spectral Efficiency

The discussion so far has considered general trends in the obtained spectra at a fixed pixel pitch, sensor thickness, and number of energy bins. The mechanisms proposed to explain these trends do not intrinsically depend on any of these factors being held constant and so are expected to be valid at other pixel pitches and sensor thicknesses. Whilst no exception to the expected order of performances is found in a random selection of spectra investigated manually in this study, the sheer number of energy spectra produced (15,400 including both normalised and non-normalised versions) means manual evaluation of all of them would be impractical. In order to reduce the task of comparing the different count-triggering schemes to a more easily automatable task, the dimensionality of the data was reduced, as described in Section 3: Results. Each energy spectrum could then be converted into two values: the spectral efficiency and the total recorded counts. The performances by these two metrics for the raw energy spectra shown so far (Figure 6, Figure 7, Figure 8 and Figure 9) were plotted against each other in Figure 13, Figure 14, Figure 15 and Figure 16. In these plots, the idealised reference point for a given system represents the highest spectral performance and count rates achievable for that system, given the limiting effects of Compton scattering, charge cloud expansion, charge trapping, and X-ray fluorescence processes. Similarly, the STD data represents the performance of the standard counting scheme employed in x-CSI systems today.

Figure 13 and Figure 14 show the performance metrics based on the spectra in Figure 6 and Figure 7, respectively (10^6^ and 10^7^ photons mm^−2^ s^−1^), again showing that the count-triggering schemes considered all perform very similarly under the low to mid X-ray flux regimes. Note that in these two figures, the y-axis does not start at 0 but rather is zoomed in to allow the schemes to be differentiated. Though the differences between best and worst performing schemes are small (~0.5 % and ~5 % for 10^6^ and 10^7^ respectively), the counting schemes can be separated into two main groups based on counts recorded: those which perform close to the reference data set (the DLR and SR schemes) and those that provide roughly the same spectral efficiency but a lower count rate (the STD, PCS, and both FDRs). Of the FDR schemes, the counting rate is marginally lower (<0.1 %) with the finite deadtime, as expected due to it being dead for some small percentage of the counts. The instantly resetting FDR, in contrast, tracks much closer to the STD and PCS cases.

These two groups diverge further as the flux increases (to 10^8^ photons mm^−2^ s^−1^, Figure 15). The FDR schemes again show very little difference to the currently employed counting scheme (STD), with roughly an 11 % reduction in counts compared with the ideal reference system. The calculated spectral efficiency of these systems appears to be slightly higher, though this is more likely to be the result of spectral distortions increasing the relative counts in the photopeak energy bins as the reference data represents the ideal performance when no such pileup is possible. DLR and PCS each appear to sacrifice one metric to preserve the other, with DLR preserving count rates whilst PCS preserves spectral efficiency. The difference in their behaviour can be understood in how each avoids negative counts. Recall that negative counts are caused when two photons arrive in such a way that some thresholds are crossed twice whilst the thresholds below them are only crossed once. In such a situation, the DLR simulates the missing crossings by incrementing the counters associated with all lower thresholds, resulting in the correct number of counts (2) after subtraction-based energy binning, though without addressing the fact that the second photon will be recorded at a higher energy. In contrast, the PCS scheme deals with this situation by preventing the thresholds crossed twice from incrementing their counters, preventing the incorrectly measured second photon from degrading the energy spectrum but at the cost of counting only 1 photon when 2 interacted with the system. An ideal compromise between these two approaches is found in the SR scheme. Here, instead of ignoring distorted counts like PCS does or fabricating new ones as DLR does, the counts recorded in the system are instead translated to the location they would have been at had the system been clear of residual charge. This leads SR to have near-ideal counting and spectral behaviour at X-ray fluxes associated with CT imaging (10^8^ photons mm^−2^ s^−1^).

The highest clinical X-ray imaging fluxes used today are around 10^9^ photons mm^−2^ s^−1^. These fluxes are associated clinically with fast imaging applications such as cardiac CT and historically are the point at which an x-CSI system’s performance drops substantially. Figure 16 shows that, at this flux level, the various count-triggering schemes are clearly differentiable in their performance, no longer being clustered together in groups. Examining the ways in which the various count-triggering schemes fail at this flux allows us to explore how important negative counts are in explaining the declining system performance at the limits of X-ray intensity.

The STD counting scheme drops to close to zero spectral efficiency and records only ~46% of the counts recorded by the idealised reference system (at this pixel pitch, sensor thickness, number of energy bins, and X-ray flux). As the FDR schemes forcibly discharge the preamplifiers after an event is counted, they are non-paralysable. The fact that they maintain approximately the same count rate as the STD scheme (instantaneous reset and 10 ns reset having <0.1 % and ~8% fewer counts respectively) thus strongly implies that the drop in count rates seen in the STD and FDR schemes are due to pileup events being summed together rather than failing to register in the system at all, as they would in the case of detector paralysis.

Turning to the PCS count-triggering scheme, we see that whilst the count rate falls almost to zero, the spectral efficiency of the counts that are recorded remain almost identical to the ideal system, markedly better than any other count-triggering scheme considered. The drop in the PCS count rate is also not due to detector paralysis, but rather occurs because only a very small number of photons arrive at a time where the residual charge in the system is below the lowest energy threshold. That detector paralysis has not occurred at this flux is also shown by the DLR counts, which performs close to the idealised count rate (~92% of REF) despite having no forced resetting architecture. Notably, however, though the spectral efficiency of DLR does drop significantly, it still performs better than the STD case, scoring roughly between the STD and FDR schemes whilst maintaining much better count rates. The improved performance compared with the STD case stems from this scheme’s complete removal of negative counts from the calculated spectrum, as it does not actually remove the excess charge which causes spectral distortions (unlike the FDR schemes). It is interesting to note that the improved spectral performance of DLR over STD scheme is arguably attributable solely to the removal of negative counts, illustrating how significant this phenomenon can be to the loss of spectral information at very high X-ray fluxes.

Finally, we consider the SR count-triggering scheme. Again, SR maintains an admirably high count rate compared with the idealised reference system (~92% of reference count rate), comparable to the DLR scheme. It has now markedly dropped in spectral performance, however, from 100 % of the idealised reference performance at 10^8^ photons mm^−2^ s^−1^ to only ~62% of the reference performance at 10^9^ photons mm^−2^ s^−1^. Despite this, SR produces a count rate comparable to DLR (which prevents negative counts causing a drop in the count rate) and a spectral efficiency higher than any other scheme except PCS (which prevents negative counts causing a drop in spectral efficiency). As noted for Figure 15, the superior count rate compared with PCS is because SR preserves spectral efficiency by attempting to correct pileup counts rather than suppressing them entirely as PCS does. Redistributing counts cannot correct for situations where the combination of residual charge and charge produced by an incident photon exceeds the highest threshold by more than one energy bin’s width, as in this situation, too few thresholds remain to be triggered above the charge floor for the energy of the photon to be correctly calculated, even if the counts are redistributed. As SR can correct for combinations of residual charge and incident photons that remain below one energy bin’s width over the highest energy spectrum, however, and as it produces a reasonable spectral efficiency, we can infer that the situation mentioned is likely relatively rare in this simulation.

### 4.4. Performance of Alternative Count-Triggering Schemes: As a Function of System Variables

To more intuitively visualise trends in the large data set collected, and to assess the adequacy of the proposed mechanisms for explaining these trends, the dimensionality of the data was reduced further as described in Section 3: Results. Plots of counts vs. spectral efficiency were produced for each simulated irradiation, with axes normalised such that the REF data point had coordinates (1,1). The distance between each count-triggering scheme and the idealised reference data in the 2D plane was then calculated, providing a single metric for overall system performance which ranged between 0 (performs as well as the idealised reference) and ~√2 (worst possible performance). By this metric, a given percentage drop in either counts or spectral efficiency is weighted equally. This may not be the case for all applications; however, the method is readily adapted by changing the relative lengths of the two axes to reflect the relative importance of each metric. Figure 17, Figure 18, Figure 19 and Figure 20 show how this metric varies as a function of the four simulated variables: pixel pitch (Figure 17), sensor thickness (Figure 18), X-ray flux (Figure 19), and number of energy bins (Figure 20). The reference system on which each parameter is varied for this study had a pixel pitch of 250 µm, a sensor thickness of 1.5 mm, an X-ray flux of 10^7^ photons mm^−2^ s^−1^ and 130 energy bins.

#### 4.4.1. Performance vs. Pixel Pitch

Figure 17 shows that the count-triggering schemes all decrease in performance with increasing pixel pitch (the distance between them and the ideal reference data increases with increasing pixel pitch). As the count-triggering scheme currently used in x-CSI is the STD scheme, the performance of the proposed count-triggering schemes relative to this is of particular interest. The non-linear shape of the STD scheme results from there being two metrics that can affect the calculated distance (spectral performance and count rate) and two mechanisms which degrade performance: pulse pileup, which increases with increasing pitch; and charge sharing, which decreases with increasing pixel pitch. The relative performance of the different schemes can also be explained with reference to these parameters.

Considering first the FDR schemes, we can divide their relative performance into three regions and the behaviour can be explained by remembering that pileup increases with pixel pitch. In the pitch range of 100–150 µm, FDR schemes do not differ significantly from the STD scheme as the time between photon interactions is sufficiently long that events are so unlikely to interact that they appear independent. Between 150 and 350 µm the time between interactions is long enough that charge can discharge between events, but sufficiently short that the finite reset time of the FDR(DT) scheme leads to counts being missed during resetting, and so the FDR(DT) scheme performs worse than the STD and FDR(Inst) schemes. Finally, above 350 µm, both FDR schemes outperform the STD scheme, though the FDR(DT) still slightly underperforms FDR(Inst). This is consistent with the average time between photon interactions now being so short that the probability of a photon interaction occurring in a pixel that has cleared its previous charge is low. In such a situation, the advantage to accurate energy estimation from forced resetting of the pixel is greater than the disadvantage of missed counts during the finite deadtime of the reset.

PCS underperforms the STD scheme for all pixel pitches except the very smallest modelled here, with the size of that underperformance initially growing with pixel pitch before reducing at larger pixel pitches, as the PCS performance tends to a fixed value. This is again explainable with reference to pulse pileup: at the lowest pixel pitches when pileup is rare, the gains in spectral performance from rejecting events where the pixel has not adequately reset are greater than the count losses from rejecting those events as the events are relatively rare. As pixel size increases, however, pileup increases and so too does the number of counts rejected, with the fractional loss in counts now outweighing the fractional gain in spectral efficiency. As the flux rate increases, PCS approaches a point where each pixel can count a single event before becoming paralysed, resulting in a high spectral efficiency but a vanishingly small count rate. At extremely high flux rates the STD scheme would also become paralysed, but its ability to count as long as the energy is below the highest bin rather than the lowest bin means it will likely generate more counts before it is paralysed at most X-ray fluxes.

The performance of the SR scheme is simple: it performs remarkably well at most pixel pitches, remaining significantly closer to the idealised reference system than the STD scheme in all cases considered here, and is almost indistinguishable in performance from the idealised counting scheme at pixel pitches below 300 µm. It does drop in performance as pitch increases, however, which can again be explained with reference to pulse pileup and the time required to clear charge. As pileup increases, so does both the average residual charge in the detector and the average change associated with a photon detection (as the probability of two photons being incident on the pixel at the same time increases). Consequently, the number of thresholds available for assessing event energy decreases (fewer thresholds are above the residual charge when a new event arrives) at the same time as the number of thresholds needed to accurately count the charge increases. This leads to the spectral performance dropping off more rapidly than the count rate, and thus the observed decrease in the overall performance.

The final scheme, DLR, also performs progressively poorer with increasing pixel pitch, again due to pileup effects causing a loss of spectral resolution as photons arrive on progressively higher residual charges. Comparing its performance to the STD scheme, DLR outperforms STD at both low (<300 µm) and high (>500 µm) pixel pitches; however, in between these pitches the STD performs marginally better than the DLR. This result does not have a simple, single mechanistic explanation; however, an intuitive understanding of what is happening can be gained by comparing Figure 14, Figure 15 and Figure 16. From these, we can see that whilst DLR loses spectral efficiency consistently from 10^7^ to 10^9^ photons mm^−2^ s^−1^. In contrast, the STD scheme initially rises in spectral efficiency (10^8^ photons mm^−2^ s^−1^) before rapidly dropping off to perform worse than DLR (10^9^ photons mm^−2^ s^−1^). As already explained, this rise in spectral performance is not a real improvement in system performance (as it moves above the ideal system), but rather an artefact of a monoenergetic irradiation: spectral distortions which shift photons to progressively higher energies will at some point inflate the number of photons appearing in the photopeak bin. The result is that the calculated spectral efficiency of DLR drops more gradually than the STD scheme but begins dropping at a lower flux. As increasing pixel pitch at a fixed X-ray flux increases the effective per pixel X-ray flux, a continuous increase in pixel pitch thus mirrors this pattern, with DLR initially performing better than STD (negative counts degrade STD but not DLR), move through a region where STD outperforms DLR (as negative counts cause artificially inflated spectral efficiencies for this monoenergetic source) and then return to a region where DLR outperforms STD (negative counts cause significant deterioration of spectral efficiency in STD, whilst DLR is still unaffected by them).

#### 4.4.2. Performance vs. Sensor Thickness

Figure 18 shows that there is only a slight negative correlation between performance and sensor thickness for all count-triggering schemes, with thinner sensors providing a slightly superior performance. At first, the small size of the effect may be surprising given that thicker pixels will result in more X-rays stopped per pixel, and Figure 17 shows that the performance of the various schemes can change markedly based on changes in per pixel X-ray flux. However, though pileup does increase with increasing sensor thickness, it increases much more slowly than with increasing pixel pitch. This is because flux increases less than linearly with sensor thickness: X-ray transmission probability decays exponentially with thickness, meaning increasing the sensor thickness by 100% (doubling it) will only increase X-ray absorption by 50%. In contrast, the number of photons intercepted per pixel increases with the square of the pixel size, meaning that increasing pixel pitch by 100% (doubling it) increases the per pixel flux by 300% (quadrupling it). As a result, in contrast to the effect of pixel pitch, varying sensor thickness has no impact on the order of count-triggering schemes when ranked by performance: SR performs best, followed by DLR, then STD and FDR(Inst), then FDR(DT), and finally PCS.

#### 4.4.3. Performance vs. X-ray Flux

Figure 19 provides an easy way to compare the performance of the various count-triggering schemes at fluxes relevant to different medical applications. At 10^6^ photons mm^−2^ s^−1^ (a flux relevant to mammography), the different counting schemes perform almost identically, which would be expected in domains in which pulse pileup is low to non-existent as the various schemes differ primarily in how they deal with pulse pileup. Even up to 10^7^ photons mm^−2^ s^−1^, there are only marginal gains (~4%) in performance in changing from the STD scheme to the best performing scheme, SR. By 10^8^ photons mm^−2^ s^−1^, however, the flux domain relevant to many clinical CT applications, there are significant differences between the proposed schemes, with SR improving performance by ~16% compared with the STD scheme, whilst PCS decreases performance by ~15% (all other schemes differ from the STD scheme by <3%). The schemes continue to diverge markedly as X-ray flux increases and, significantly, by 10^9^ photons mm^−2^ s^−1^, all newly proposed count-triggering schemes perform better than the STD scheme currently employed, with improvements of between ~42% (PCS) and ~246% (SR) seen. The order of performance of the various schemes does not change at the fluxes considered, only with respect to the STD scheme.

It should be remembered that the pixel pitch and sensor thickness used in generating Figure 19 were specifically chosen to maximise the performance of the STD scheme under the monoenergetic irradiation performed here, based on previous work [17,20]. For that reason, it is perhaps not surprising that STD was only clearly inferior to all other schemes at the highest fluxes investigated and may seem obvious that changes to the pitch or thickness could shift the point at which STD becomes the worst scheme. However, even in the highest per pixel flux case considered here (3 mm thick sensor, 600 µm pixel pitch), the PCS scheme underperforms the STD scheme at all but the highest X-ray flux considered. The result that PCS is inferior to the currently used STD scheme at all but the highest medically relevant fluxes thus seems robust, as does the observation that at most fluxes, the SR scheme performs the best.

#### 4.4.4. Performance vs. Number of Energy Bins

Finally, Figure 20 shows how count-triggering scheme performance varies as a function of the number of energy bins used. It is important to remember that the changes in performance are shown with respect to the idealised reference system and are thus not comments on absolute performance. It appears that for most schemes considered (STD, DLR and both FDRs) there is an optimal number of energy bins at which their performance is maximised, with this value being lower for the DLR scheme (5 energy bins) than the others (24 energy bins for the STD and FDRs). The shape of these dependencies is reliable across a wide range of data sets chosen at random from the simulated systems: FDR and STD schemes have a clear bowl shape, DLR has a similar shape, however often with a sharp localisation of the peak performance, and both SR and PCS show a near flat response. The sharp local dip in the DLR data at 5 energy bins is unexpected and is not easily explained by the mechanistic explanations given thus far. A computational anomaly in a single data set was ruled out after noting that this feature is still present over a wide range of X-ray fluxes, pixel pitches and sensor thicknesses. Identifying the causes of this dip would require further simulation work, with more energy bin numbers near to this peak (e.g., 4, 5, 6, and 7), to evaluate the nature of the change observed. This is beyond the scope of the current investigation, though may be the topic of future work, should the DLR scheme prove popular/applicable for a specific application.

PCS and SR are different from the other schemes in that they actively resist the residual circuit charge from shifting new photons into an artificially high-energy bin: PCS does not count if the residual charge is above the lowest threshold and SR subtracts an estimate of the residual charge from the calculated energy. As these two schemes count as if there is no residual charge in the system, their performance varies in proportion with the idealised reference, in which no system charge is allowed to build up, and so their lines appear flat.

In contrast, STD makes no correction for residual charge, FDR schemes periodically remove residual charge (but behave as STD in between resets), and the DLR scheme prevents negative counting but still records the combined photon + residual energy as the counted energy. As a result, the performance of these schemes does depend on the residual charge in the system at the time of a second photon interaction. The bowl shape with a local minimum implies at least two mechanisms: one which causes performance to gradually decline with increasing number of bins and one which makes it more sharply decline at the lowest numbers of energy bins.

The decrease in performance at low numbers of thresholds can be explained by a drop in the counting rate at low numbers of thresholds. As the number of thresholds increases, the width between them increases. For an idealised counting scheme this will not matter as each event is compared with the lowest threshold and registers a count if it is above the noise floor of the system (>10 keV for <130 energy bins). For schemes which do not correct for residual charge between events (STD, FDR and DLR), however, a wider gap between bins means that it is more likely that a second photon arriving will not increment a counter, as the gap between the residual charge and the next threshold is greater than the energy it deposits. At 5 bins, the width is 30 keV, meaning counts from X-ray fluorescence (<30 keV) may not trigger a count if they pile up on a residual charge just over threshold, whilst by 3 bins the width is 60 keV, meaning both X-ray fluorescence and escape peaks (<60 keV) could fail to be counted. The result is that at these low numbers of energy bins, more photons are missed and so the counting performance of the STD, FDR, and DLR schemes is reduced sharply.

The more gradual decrease in performance seen at higher numbers of energy bins can be explained by a drop in spectral efficiency as the number of energy bins increases. This initially may seem counterintuitive as larger numbers of energy bins provide more fine spectral information; however, it needs to be remembered that the drop in performance is relative to an ideal counting scheme, not an absolute drop. There may indeed be a greater spectral performance at larger pixel pitches; however, these gains are realised more slowly for the counting schemes examined than for an ideal counting scheme. The spectral efficiency would decline with increasing number of energy bins because more energy bins means:

1. the energy of incident photons is more likely to be located near to a threshold above them, making it easier for the residual charge from a previous event to push the signal into the wrong energy bin;
2. the signal produced from any event (residual charge plus photon induced charge) is more likely to be located near to a threshold below it, reducing the minimum time between photons needed before a negative count can be caused.

As DLR is not able to produce negative counts, it is not affected by the second of these two points, justifying why its optimal performance may lie at a different number of energy bins.

Combined, the drop in the spectral efficiency with an increasing number of bins and the drop in the count rate at the lowest numbers of bins produce the bowl-shaped curves with local maxima seen in Figure 20 for the STD, DLR, and FDR schemes.

### 4.5. Caveats, Limitations and Further Work

**Figure 23:**
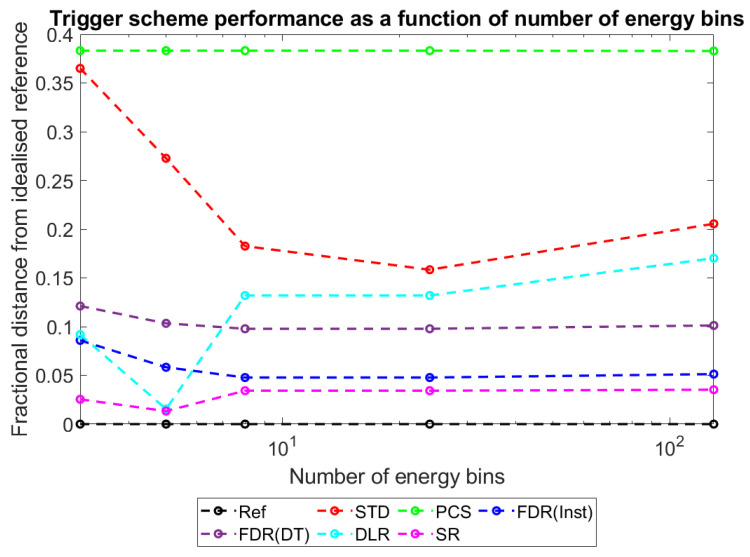
Plot of count scheme performance as a function of number of energy bins. Parameters were the same as for Figure 20, with the exception of the reset delay in FDR schemes (10 ns here vs. 100 ns in Figure 20) and the integration time of the digital adder in the SR scheme (10 ns here, 1 ns in Figure 20).

The counting schemes simulated in this work necessarily required assumptions be made regarding how they would be implemented in a circuit, as they are not currently employed in physical circuitry. Timing parameters were chosen which are consistent with other systems with which the authors have experience; however, as the proposed novel counting schemes have yet to be constructed physically, it remains to be seen if the estimated timing characteristics are technically feasible. As a test to show how sensitive the observed ordering of count-triggering schemes was to these timing parameters, a second set of simulations was performed with more favourable timing characteristics for PCS (1 ns AND gates with instant signal rise times) and FDR (10 ns delay before resetting, still a 10 ns deadtime for that model) and less favourable timing characteristics for the SR scheme (10 ns integration of events in the digital adder before writing out). These modifications made the PCS and FDR schemes less susceptible to pulse pileup, whilst simultaneously reducing the performance of the SR scheme. As can be seen in Figure 23, despite the order of magnitude improvement in timing for PCS and FDR, and the simultaneous order of magnitude decrease in timing resolution for SR, the relative order of PCS, FDR, and SR schemes is not changed in most simulated systems. FDR and SR performance did become much closer, however, and in a few systems, FDR(Inst) outperformed SR, though FDR(DT) still did not. Importantly, the FDR schemes show a significantly reduced curvature in their response, which supports the assertion made previously that the curvature in the FDR and STD schemes results from pulse pileup as the reduction in their reset window will have had the primary effect of reducing pileup.

With a small number of exceptions, the faster FDR schemes modelled perform better than the DLR scheme. The relative ordering of PCS, STD, FDR, and SR by performance was, however, demonstrated to be robust to significant changes in the timing parameters used, providing confidence that the general trends in performance will hold for a wide range of timing parameters that could be employed in constructing these schemes in physical circuits.

A major limitation of the SR count-triggering scheme which has not previously been discussed is that it requires the spacing between adjacent energy thresholds to be held constant. This is in contrast with all other schemes considered here which allow thresholds to be adjusted arbitrarily. Consequently, the SR scheme may be limited in its ability to exploit certain benefits associated with the free selection of energy bins, such as ensuring statistically significant numbers of counts are recorded in each bin, or gating around several closely spaced k-edges for multi-contrast imaging. Whether or not these limitations are sufficient to offset the significant advantages of this technique will depend on the specific application envisaged. The data presented here suggest that the benefits are unlikely to outweigh these costs up to at least 10^7^ photons mm^−2^ s^−1^, as the various schemes all perform similarly up to this flux. It also remains to be seen how rapidly the performance of the SR scheme declines as bin widths become unequal. Determining for which applications the SR scheme remains best, and for which the FDR or DLR would be better, will thus require more detailed, task-specific simulations in the future.

## 5. Conclusions

The work presented here comprises two parts. First, the existence of a previously undescribed phenomenon in X-ray photon counting spectral imaging (x-CSI) systems, which we refer to as negative counting, is demonstrated. Negative counts are caused by pulse pileup but they degrade spectral performance in a different way to pulse pileup in other spectroscopic systems: in addition to producing a pileup count, a count is also subtractive from the nearest threshold below the level of residual charge when the pileup event arrived. The rarity of negative counts is correlated to how close residual charge lies to a threshold, meaning the probability of negative counts increases with an increasing number of thresholds. This, combined with the fact that higher energy photons are normally vastly outnumbered by low-energy photons, is proposed as a reason why negative counts have not been observed experimentally to date. Simulation results are presented to show that negative counts would manifest as a reduction in low-energy bin counts, rather than negative values for those counts, when the number of energy thresholds is in the range currently employed in x-CSI ASICs.

Demonstrating the existence of, and elucidating the mechanism behind, negative counts is an excellent example of the powerful role computational tools can have in detector design and prototyping. It would not have been possible to empirically demonstrate the existence of this phenomenon with existing physical systems given both the technical feasibility and financial constraints associated with building the wide range of systems simulated.

The second part of the work presented concerns an investigation of four new count-triggering schemes which we have proposed to alleviate the detrimental effects of negative counts. These schemes are compared with the currently employed counting scheme, referred to as STD in this work, for their spectral efficiency and counting accuracy. The performance of each scheme is compared with an idealised counting scheme, and the relative performance is calculated using an equal weighting of spectral performance and count efficiency. This process is repeated for 1100 different combinations of pixel pitch (100–600 µm in 50 µm steps), sensor thickness (1–3 mm in 0.5 mm steps), number of energy bins (3, 5, 8, 24 and 130), and X-ray fluxes (10^6^, 10^7^, 10^8^ and 10^9^ photons mm^−2^ s^−1^). These data are used to establish how each scheme affects the system performance as a function of pixel pitch, sensor thickness, number of energy bins, and X-ray flux.

At X-ray fluxes below 10^8^ photons mm^−2^ s^−1^, there is little difference between the different schemes investigated. At this X-ray flux and above, of the four proposed alternative schemes, PCS preserves spectral performance the best; however, it drops in count efficiency so rapidly that its overall performance is less than STD in almost all cases. In contrast, the SR scheme outperforms all of the others, maintaining an almost ideal response in many simulations. FDR and DLR schemes both outperform the STD scheme, with their relative ordering dependent on timing parameters chosen for the FDR scheme.

The results of these simulations suggest that significant gains in both spectral efficiency and counting performance can be obtained by adopting one of the alternative count-triggering schemes proposed in this work, other than PCS.

## Figures and Tables

**Table 1:** List of parameters used in the Monte Carlo simulations in Component 1.

Parameter	Value	Unit
X-ray source type	Monoenergetic	-
X-ray energy	80	keV
Irradiation type	Flatfield	-
Sensor material	CdTe	-
Sensor density	5850	kg m^−3^
Sensor cross-sectional area	21 × 21	mm^2^
Sensor thickness	1 to 3 (steps of 0.5)	mm

**Table 2:** List of material properties used in the Finite Element Method simulations in Component 2.

Parameter	Symbol	Value	Unit
Mobility, electrons	µ_e_	1100	cm^2^ V^−1^ s^−1^
Mobility, holes	µ_h_	100	cm^2^ V^−1^ s^−1^
Lifetime, electrons	τ_e_	3.0	µs
Lifetime, holes	τ_h_	2.0	µs
Density	Ρ	5850	kg m^−3^
Diffusion coefficient, electrons	D_e_	2.84 × 10^−3^	m^2^ s^−1^
Diffusion coefficient, holes	D_h_	2.58 × 10^−4^	m^2^ s^−1^
Relative permittivity	ε	11.0	-
Pixel pitch	S_P_	100 to 600 (in steps of 50)	µm
Sensor thickness	S_T_	1 to 3 (steps of 0.5)	mm

**Table 3:** Summary of the timing parameters used in modelling the count-triggering schemes.

Parameter	Value
Signal decay model	Exponential
Decay time (to 1%)	100 ns
AND gate open time (PCS only)	10 ns
Reset delay (FDR(Inst) and FDR(DT))	100 ns
Reset deadtime (FDR(DT) only)	10 ns

**Table 4:** Summary of the count triggering schemes modeled.

Acronym	Full Name	Quick Description
STD	Standard approach	Each threshold is linked directly to its corresponding counter.
PCS	Premature Count Suppression	Higher counters can only be incremented if the lowest counter was recently incremented.
FDR	Forced Delay Reset	A fixed time after the lowest threshold is crossed, the charge on the anode is reset to zero.
DLR	Descending Line Responder	When a counter is incremented, any untriggered counters below it are also incremented.
SR	Shift Register	The number of thresholds recently crossed is counted and then distributed across counters, starting from the lowest to the highest.

**Table 5:** List of the bins which constitute *Np* in Equation (3).

Number of Energy Bins Simulated	Bin Numbers Containing Photopeak
3	2
5	3
8	5
24	13–15
130	70–81

**Table 6:** Conversion from number of times a threshold is crossed to the counts recorded in that energy bin shows how negative counts can originate from the data shown in Figure 21.

Threshold–Counter Pair	Counts	Energy Bin	Calculated Counts
1 (1.5 keV)	1	1.5–10 keV	0
2 (10 keV)	1	10–18.5 keV	−1
3 (18.5 keV)	2	18.5–27 keV	1
4 (27 keV)	1	>27 keV	1

## Data Availability

Not applicable.
